# Full scale structural, mechanical and dynamical properties of HIV-1 liposomes

**DOI:** 10.1371/journal.pcbi.1009781

**Published:** 2022-01-18

**Authors:** Alexander J. Bryer, Tyler Reddy, Edward Lyman, Juan R. Perilla

**Affiliations:** 1 Department of Chemistry and Biochemistry, University of Delaware, Newark, Delaware, United States of America; 2 CCS-7 Applied Computer Science, Los Alamos National Laboratory, Los Alamos, New Mexico, United States of America; 3 Department of Physics and Astronomy, University of Delaware, Newark, Delaware, United States of America; Icahn School of Medicine at Mount Sinai, UNITED STATES

## Abstract

Enveloped viruses are enclosed by a lipid membrane inside of which are all of the components necessary for the virus life cycle; viral proteins, the viral genome and metabolites. Viral envelopes are lipid bilayers that adopt morphologies ranging from spheres to tubes. The envelope is derived from the host cell during viral replication. Thus, the composition of the bilayer depends on the complex constitution of lipids from the host-cell’s organelle(s) where assembly and/or budding of the viral particle occurs. Here, molecular dynamics (MD) simulations of authentic, asymmetric HIV-1 liposomes are used to derive a unique level of resolution of its full-scale structure, mechanics and dynamics. Analysis of the structural properties reveal the distribution of thicknesses of the bilayers over the entire liposome as well as its global fluctuations. Moreover, full-scale mechanical analyses are employed to derive the global bending rigidity of HIV-1 liposomes. Finally, dynamical properties of the lipid molecules reveal important relationships between their 3D diffusion, the location of lipid-rafts and the asymmetrical composition of the envelope. Overall, our simulations reveal complex relationships between the rich lipid composition of the HIV-1 liposome and its structural, mechanical and dynamical properties with critical consequences to different stages of HIV-1’s life cycle.

## Author summary

Lipid membranes are a critical component of enveloped viruses such as HIV-1. Derived from the host cell, the viral envelope serves as a foundation for membrane proteins and a container for the viral payload, facilitating every stage of the viral cycle and ensuring proliferation. Here, we present simulations and analysis of an authentic HIV-1 liposome to better understand its mechanical and dynamical properties, as well as to investigate the interplay between constituents of its complex lipidome. Further, simulations of a flat control system allow us to probe the effects of curvature on lipid dynamics and phenomena such as transverse bilayer lipid diffusion.

## 1 Introduction

Enveloped viruses are molecular pathogens enclosed in a lipid bilayer acquired from the host cell during viral assembly and egress [1]. The lipid envelope and membrane proteins act as a container for all the components necessary to fulfill the virus life cycle [2], adopting morphologies ranging from spheres to tubes [3]. The lipid bilayer itself is derived from the host cell during viral replication. Thus, the composition of the bilayer depends on the complex constitution of lipids from the host-cell’s organelle(s) where assembly and/or budding of the viral particle occurs [4–7]. The human immunodeficiency viruses type 1 (HIV-1) is an enveloped retrovirus that infects immune system cells, specifically CD4^+^T cells and macrophages [8, 9]. Initially, the virion enters the host cell by direct fusion with the plasma membrane, facilitated by viral membrane proteins [10, 11]. During the late-stages of the life cycle, assembly of the virion inside the host-cell takes place at the plasma membrane directed by specific lipid-protein interactions [8, 9, 12].

Budding and assembly of the viral particle takes place at specific locations on the plasma membrane (PM) [5]. Essential to viral fitness is the structural Gag polyprotein which regulates several events during viral replication [9]. For instance, anchoring of HIV-1’s Gag to the inner leaflet drives viral particle assembly at specific microdomains of the PM [9, 13]. More specifically, the anchoring of Gag to the PM is driven by the matrix domain (MA). MA contains a myristate moiety, that in the presence of phosphatidylinositol 4,5-bisphosphate (PIP_2_), facilitates the insertion of the acyl chain to the inner monolayer, inducing Gag assembly in PIP_2_-rich domains [14, 15]. Moreover, the specific constitution of lipids plays a role in membrane deformation and curvature of the vesicle during particle budding [16], and the stiffness of the virion is an essential physical determinant for HIV-1 infectivity related to specific life-stages of the virus [17–19].

As previously metioned, HIV-1 virions are composed of proteins, metabolites, sugars and lipids [20]. High precision characterization of the lipidome of authentic HIV-1 virions has been reported by lipidomic analyses [19, 21, 22]. In addition to sphingomyelin (SM) and cholesterol, the HIV-1 lipidome contains a high percentage of phosphatidylserine (PS) and phosphatidylethanolamine (PE) [18, 19, 21]. Therefore, it was initially suggested that the lipid composition of the viral lipid vesicle resembles that of a typical mammalian plasma membrane, with a prevalence of phosphatidylcholine (PC) and SM in the outer leaflet, and PS and PE lipids in the inner leaflet [23]. However, differences in the composition of the lipid vesicle and the plasma membrane have been observed, indicating that viral morphogenesis occurs on lipid microdomains of host-cells [7, 9, 22, 24].

**Fig 1:**
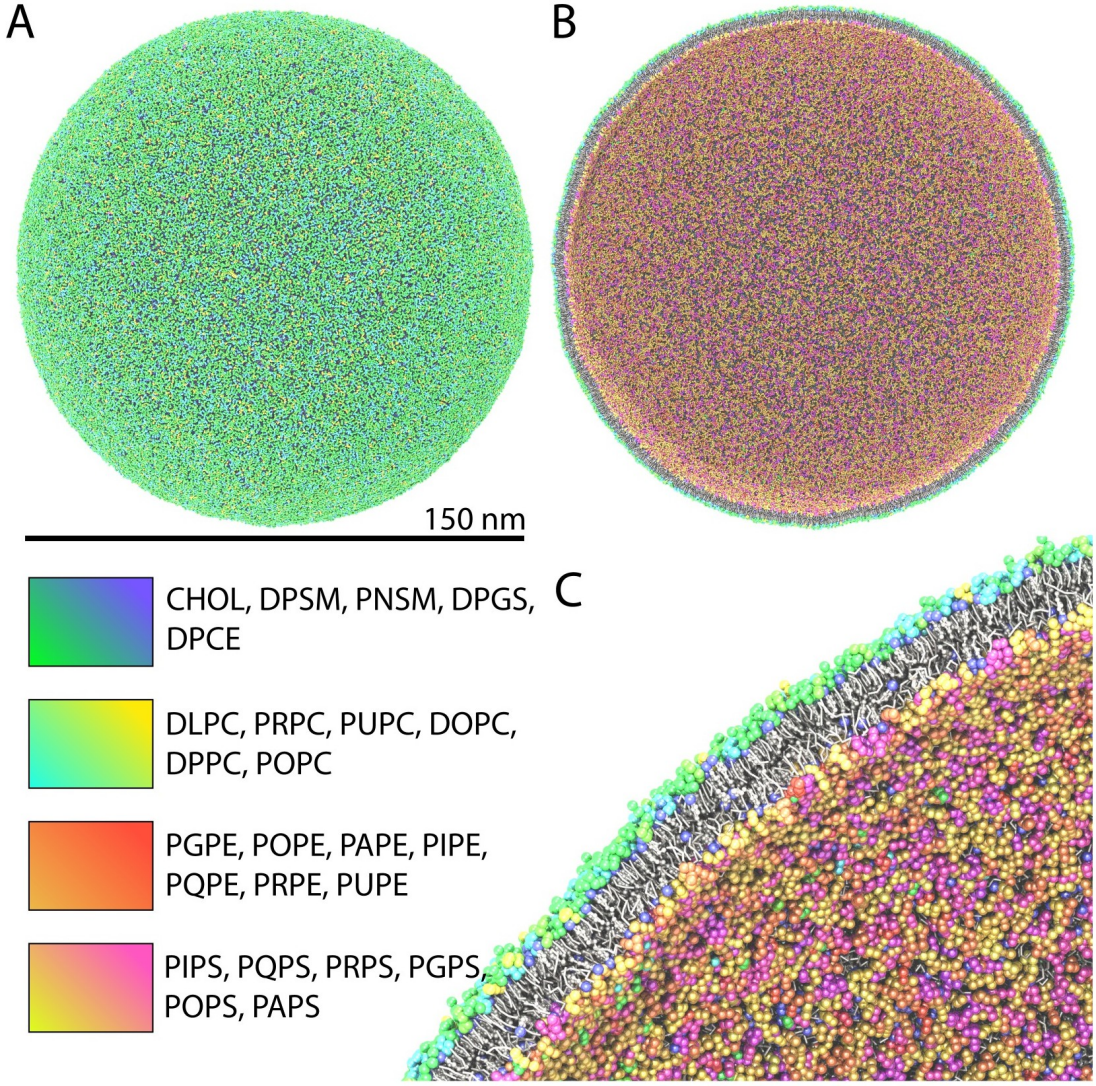
Full-scale model of a realistic HIV-1 lipid vesicle at united atom resolution (MARTINI force-field). **A** Full-view representation of the 150 nm vesicle model. **B** Clipped view of the vesicle and **C** close-up view of the clipped vesicle, the latter demonstrating lipid packing across the bilayer. In each panel, the headgroup of each chemical species is colored differently according to the legend provided. Tails are shown in licorice representation, and Cholesterol is shown in ball-and-stick representation.

Although recent advances in experimental techniques allow the characterization of structural properties of entire viral particles, molecular dynamics (MD) simulations provide a unique level of resolution for full-scale virion models and large-scale macromolecular complexes [25–28]. Here, we present a full-scale model of the HIV-1 liposome with physiological lipid composition derived from lipidomics of infective HIV-1 virions. Structural and dynamical characterization of an authentic HIV-1 liposome were performed using massively parallel computing [29], over 5.2 *μ*s of simulation. The model of the HIV-1 vesicle (Fig 1) includes 24 distinct lipid-species (S1 Fig) with a composition asymmetrically distributed in the inner and outer leaflets (S1 and S2 Tables). In addition to the full-scale vesicle model, we constructed and simulated a 46 x 46 nm flat membrane patch with identical lipidome specifications as a control.

Despite significant flip-flop of lipids between the leaflets of the full-scale vesicle, the asymmetric composition is maintained within tight tolerances over the course of the simulation, suggesting a link between asymmetry and the shape of the envelope, even in the absence of envelope proteins. Supporting the role of envelope shape in lipid flip-flop, we observed no transverse bilayer diffusion events in our flat membrane control system. Analysis of the structural properties reveals the global fluctuations of the bilayer and of the bilayer thickness. The bending rigidity of the liposome is obtained from an analysis of fluctuations in its shape, while for the flat patch control system, we obtain compressibility moduli through fluctuations in thickness. We also find a heterogeneous organization of lipids into nanoscale ordered domains, with correspondingly heterogeneous dynamics, in both the full-scale vesicle and flat patch systems. We anticipate that our results will guide the simulation of entire viral particles including membrane proteins, viral proteins and the viral genome; furthermore, the analysis framework derived herein can be readily applied to other enveloped viruses.

## 2 Results

A full scale model of an HIV-1 liposome was built, equilibrated for 1.5 *μ*s and subsequently simulated for 5.2 *μ*s using the MARTINI force-field [30] (S1 Movie). A rhombic dodecahedron solvation container was chosen to minimize the volume of the simulation system as shown in Fig 1A. The liposome was constructed to have an initial outer diameter of 150 nm (Fig 1B), falling in the range of outer lipid diameter values reported experimentally [31]. The overall composition of the lipid vesicle was chosen to capture the chemical diversity of lipids observed by lipidomic analyses of infective HIV-1 virions (S1 Table) [19, 21, 22]. The initial distribution of lipids across the two leaflets is not known precisely for the HIV-1 virion, thus it was chosen based on well known plasma membrane asymmetry: negative charge, unsaturation, and ethanolamine on the inner leaflet; sphingolipids on the outer leaflet [24]. Utilizing MARTINI [30] and the same asymmetric lipidome profile, we constructed and simulated a flat HIV-1 membrane patch. The latter acts as a control and provides insight into the effect of envelope shape, i.e., curvature, on the various physical properties of the membrane. From the simulations, a wealth of biophysical properties were determined for an authentic viral liposome as presented in the following sections.

### 2.1 Bilayer thickness and liposome sphericity

The outer diameter and sphericity of the HIV-1 lipid vesicle was measured over the first 1.5 *μ*s of equilibration. Convergence is observed for both parameters, with an average of 145 nm and 0.9975 for the outer diameter and the sphericity, respectively (S3 Fig). During production, an increase of the outer diameter from 145 nm to 147 nm is observed. Sphericity is maintained with an average of 0.9984 throughout 5.2 *μ*s production sampling (S4 Fig).

**Fig 2:**
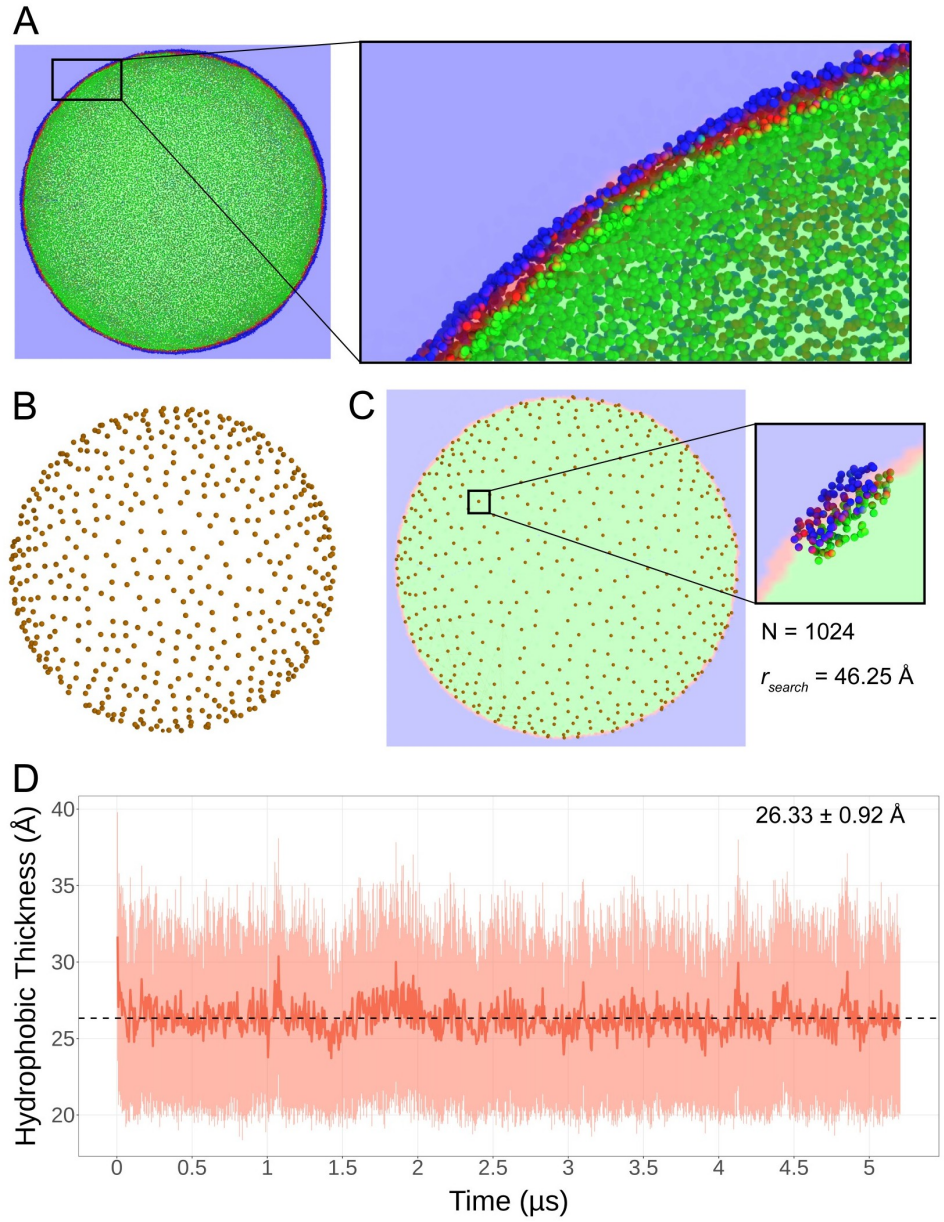
Hydrophobic thickness analysis. **A** Clipped view of the carbon bead selection used for thickness calculation. The selection is superimposed with the volume grid employed to determine the leaflet in which each ‘C2B’ bead resides; blue represents the outer monolayer and green the inner monolayer. The inset shows a zoomed in view of the leaflets delineated by the volume grid, the latter yielded by *measure volinterior*. **B** The uniform point distribution (*N* = 1024) used to sample thickness, yielded via Poisson disk sampling. **C** Uniform distribution with the volume grid. The inset demonstrates the carbon beads isolated about each test point, in a search radius *r*_*search*_ of 46 Å. Hydrophobic thickness is considered as the center of mass distance between groups of C2B beads in each leaflet. **D** Resulting analysis over the 5.2 μs trajectory. The dark line shows the mean hydrophobic thickness per frame, while the transparent region shows the standard deviation at each frame for the 1024 values calculated. The mean thickness over the time series, dotted line, is 26 Å with a standard deviation of 1 Å between frames.

The first step in computing the hydrophobic thickness was to delineate interior and exterior regions of the vesicle system, such that ‘C2B’ beads (corresponding to carbons C08:0-C12:0) of lipids in the membrane could be classified based on their leaflet of residence (Fig 2A). Calculation of hydrophobic thickness was obtained from *N* = 1024 uniformly distributed regions of the vesicle (Fig 2B), the positions determined with a Poisson disk sampling technique [32]. About each point in the sample distribution, C2B beads were selected within a search radius of 46.25 Å (Fig 2C), and the center of mass distance between beads in the inner and outer leaflets was computed.

Utilizing a high-performance analysis framework outlined previously [29, 32], built on VMD with MPI support, we analyzed the 5.2 μs trajectory (Fig 2D). The average hydrophobic thickness for the time series is 26 ± 1 Å. As shown in the plot, fluctuations of thickness computed in this manner, where the computed value for each frame is a mean of 1,024 samples distributed evenly across the vesicle surface, are relatively small at less than 1 Å. The volume grid used to classify space around the vesicle was computed at intervals of 125 ns throughout the trajectory. The analysis utilized 42 MPI ranks, each of which computed the volume grid with its first frame before analyzing its 125 ns component of the complete time series. Frames utilized for *measure volinterior* space classification were discarded from analysis to remove an artifact causing thickness to be underestimated.

An additional analysis of overall membrane thickness was carried out in a similar manner. Center of mass distances between head groups in each leaflet were calculated across uniformly distributed regions of the vesicle surface. The values were found to fluctuate within 34 to 36 Å. The observed overall thickness and subsequently small variations is in agreement with observations made by cryo-electron tomography for vesicles with a 42% molar ratio of cholesterol [33]. No relationship between local thickness values and composition was found in our analyses.

### 2.2 Transmembrane asymmetry

**Fig 3:**
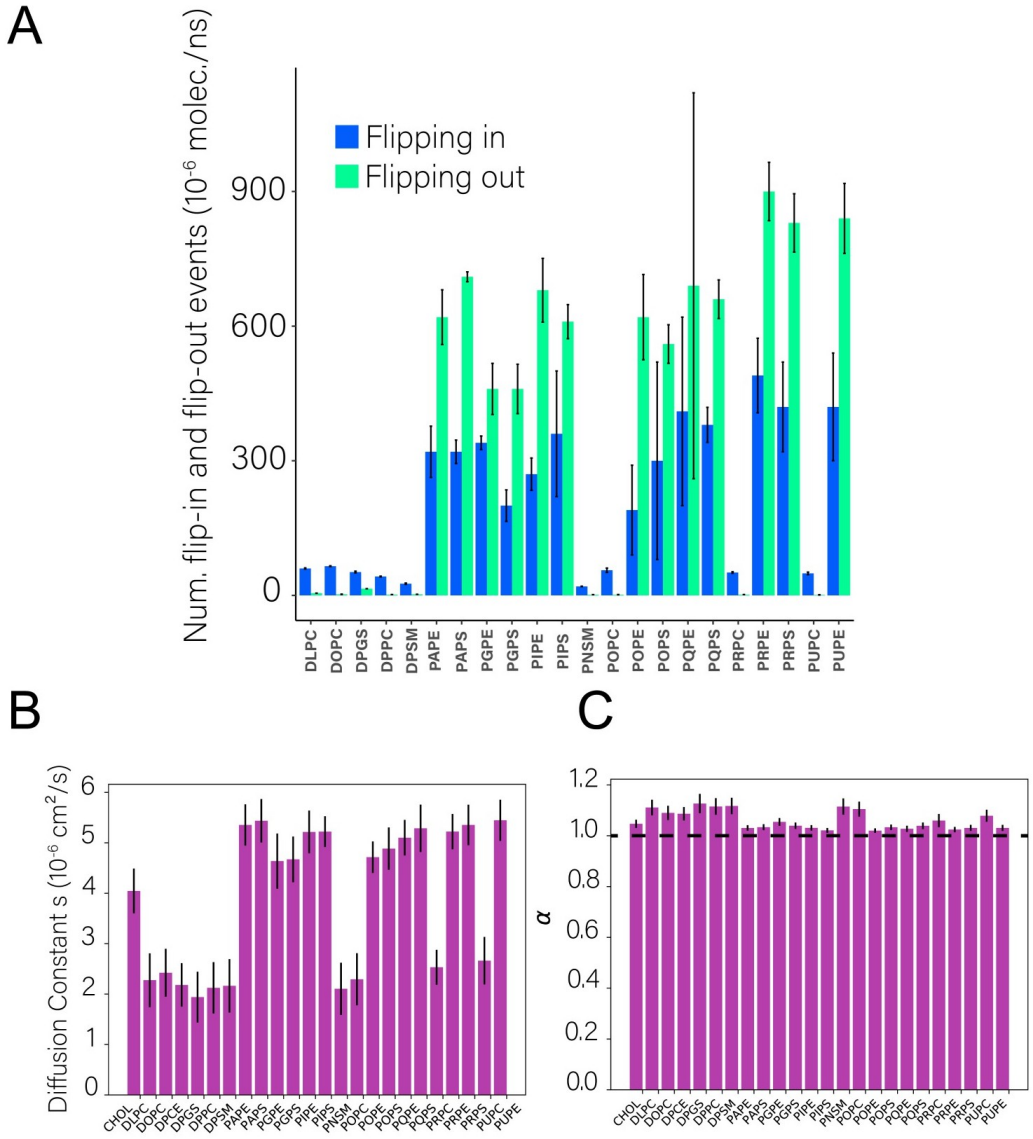
Diffusion of lipids on the complex vesicle model. **A** Trans-bilayer rates of lipids present in the vesicle after 5.2 *μ*s of MD production. The average outer to inner rate (blue) and outer to inner (red) rates were estimated by calculating the cumulative number of flipping events per lipid species from three different starting points during the simulation: 0, 200 and 4,000 ns. The values reported are normalized per number of events per molecule. **B** Lipid lateral diffusion coefficient and **C** scaling factor. The average is reported at windows sizes of 25, 50, 100, 250, 500 and 1,000 ns.

Significant diffusion of lipids between the bilayer leaflets (“transverse” diffusion) is observed throughout the full-scale vesicle simulation (Fig 3, S5 Fig; S2 Movie). To characterize the transverse diffusion of lipids, flip (outer-to-inner) and flop (inner-to-outer) rates were calculated separately during the 5.2 *μ*s of MD production for each lipid species. Previous MD simulation on flat-patch asymmetric membranes suggest the flip and flop rates are slow processes in the second timescale, supported by experimental small-angle neutron scattering (SANS) results [34]. The rate of flip and flop are influenced by the chain length, temperature and chemical character of the lipid headgroup.

Average flip-flop rates were calculated for each lipid species over the entire production simulation of the HIV-1 liposome (Fig 3A); transbilayer flip-flop rates over different portions of the simulation are shown in S5 Fig). There are clearly two classes of lipids in Fig 3A: lipids with rates less than 100(10^−5^) events/ns × molecule, and lipids with rates higher than 200(10^−5^) events/ns × molecule. The first category corresponds exclusively to lipid types that are enriched in the outer leaflet (sphingolipids and saturated chain lipids), while the second is exclusively lipids enriched on the inner leaflet (highly unsaturated lipids with PE and PS headgroups). Although the flip-flop rates are slow (as expected for lipid transbilayer rates), they do represent a substantial lipid translocation in the aggregate for many lipid types. For example, the total number of PAPS inner→ outer events is 40, while the total number of outer → inner events is 15. In spite of this, the initial and final numbers of PAPS in each leaflet are extremely asymmetric, and maintained within ± 2 lipids. Indeed, the total number of flipping events is larger than the number of PAPS in the outer leaflet. This is true for many of the lipid types, and indicates (i) that the difference in flip flop rates observed between inner and outer leaflets is not due to directional transport as the composition equilibrates by flip flop, and (ii) that compositional asymmetry would be maintained over longer timescales. Given that the composition of the two leaflets has not been experimentally determined, it is remarkable that the initial asymmetry is maintained during the *t* = 5.2 *μ*s of MD production, despite significant rates of lipid flip flop.

Given the observed stability of lipidomic asymmetry, the leaflet compositions reported in S8 Fig yield a good model for an HIV-1 lipid vesicle. These results are not only in agreement with bulk lipidomics results for the HIV-1 virion [21], but are also supported by lipidomics experiments that suggest the formation of lipid domains on the host-cell, where budding and release of the viral particle takes place [35, 36].

Interestingly, transbilayer diffusion of phospholipids in the flat HIV-1 membrane patch was not observed, and the lipid compositions of each leaflet are maintained throughout the entirety of our simulations. This result further suggests a relationship between curvature and lipid flip flop.

### 2.3 Lateral diffusion of lipids is heterogeneous within and between the leaflets

Characterization of the lateral diffusion of lipids was performed using methods previously developed by the authors for the analysis of full-scale virions [27]. Analysis of time- and ensemble-averaged lipid mean squared displacements (MSD) show the diffusion to be Fickian (not subdiffusive). The diffusion constant (*D*) for different lipids in the inner and outer leaflets was obtained from fits of the MSD (Fig 3B), with all diffusion constants on the order of 10^-6^ cm^2^/s. Lateral diffusion of lipids in the inner and outer leaflets of the flat HIV-1 system shows the same trend (S11 Fig), where MSD of the inner leaflet is substantially higher than that of the outer leaflet.

**Fig 4:**
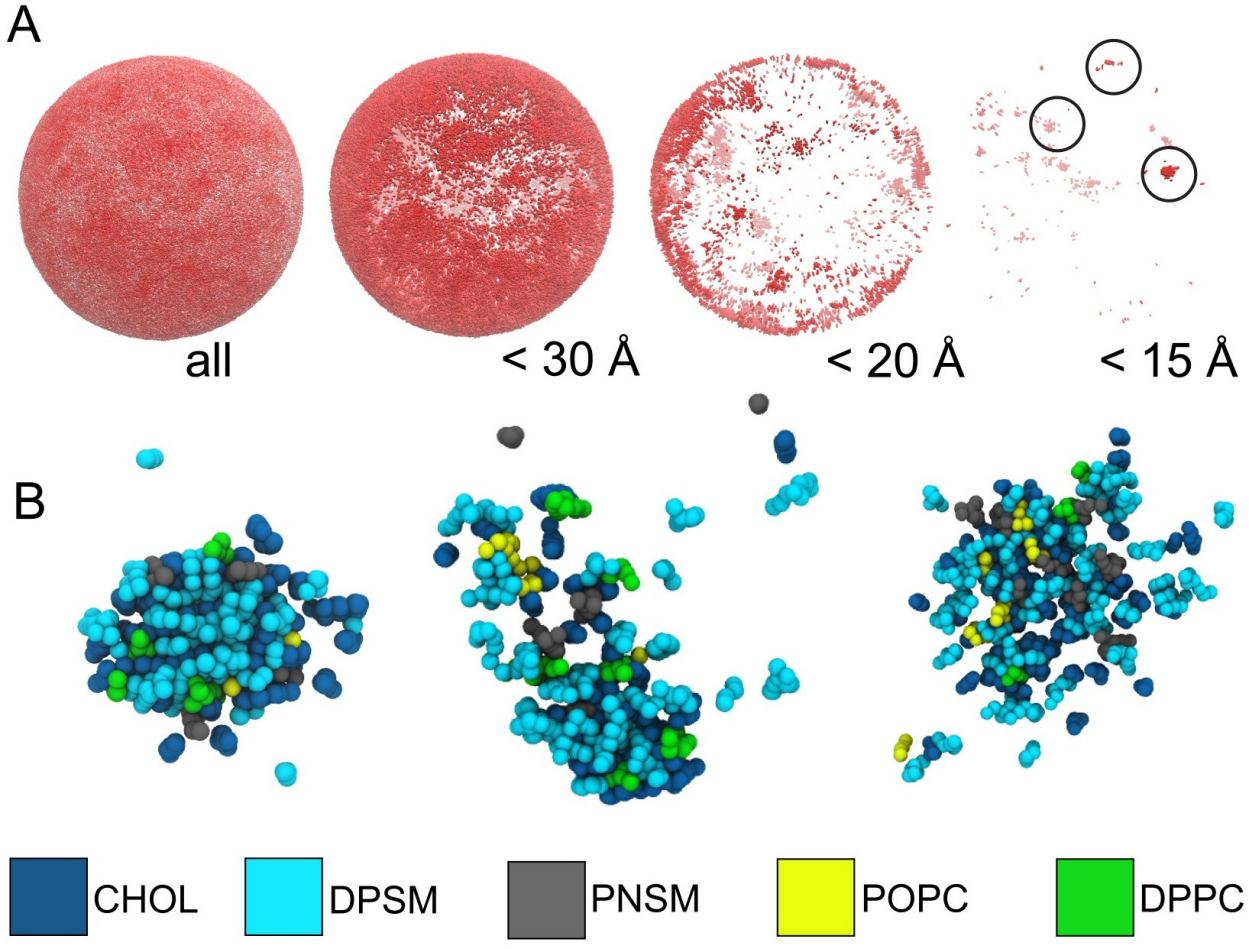
Mobility analysis of lipids and lipid regions. **A** Mobility of lipid headgroups after 1 μs of production MD, in the outer leaflet. Each column shows lipids with decreasing mobility cutoffs, down to 15 Å. **B** Visualization of the low-mobility domains, circled in panel A, below 15 Å mobility. Chemical species are colored according to the legend provided.

Upon visual inspection of the trajectories it is clear that there are lipid domains on the vesicle that displace laterally much more slowly than the surrounding lipid matrix (S3 Movie). To characterize these lipid regions based on their lateral mobility, the root-mean-squared fluctuations were calculated for each lipid molecule over different windows of the final 500 nanoseconds of the production simulation (Fig 4). In Fig 4A, several localized domains of low mobility lipids (dark patches) are observed, with diameters of roughly 10 nm. No such domains are observed on the inner leaflet. A further characterization of some of the most dramatic low-mobility domains on the outer monolayer reveals that these domains are enriched in cholesterol, sphingomyelin and phosphatidylcholine (Fig 4B).

The low mobility domains remain intact for a few nanoseconds and subsequently diffuse over the surface of the vesicle—they are transient, fluctuating domains which appear to be intrinsic to the composition of the outer leaflet of the lipid vesicle. During the simulation these low-mobility regions appear and disappear, highlighting their dynamic nature (S3 Movie). Importantly, these low mobility regions are observed only in the outer leaflet and completely absent in the inner leaflet (S10 Fig).

### 2.4 Elastic properties

**Fig 5:**
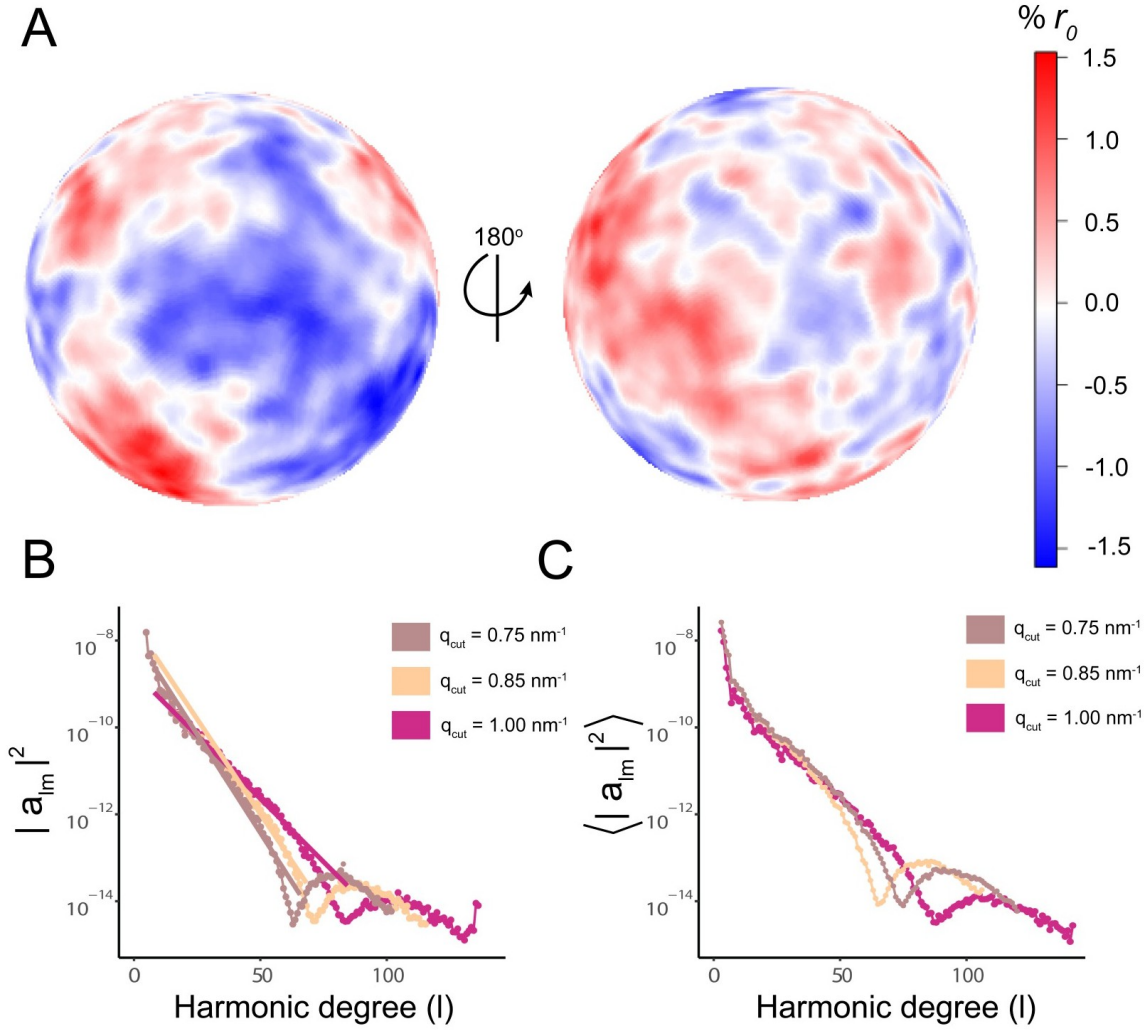
Bending rigidity of the HIV-1 lipid vesicle. **A** Undulating surface of the radial fluctuations as obseverd after 5 μs of MD production. Units of fluctuations are given as percent of the average radius. **B** Undulation spectra from the expansion in spherical harmonic of the undulating surface for a single simulation snapshot of the liposome; the fit is shown for wave-number *q* cutoffs [38] of 0.75 nm^-1^, 0.85 nm^-1^ and 1 nm^-1^. A linear fit is shown for the initial l-degrees for the three different cutoffs. **C** Ensemble average undulation spectra of the HIV-1 liposome. The bending rigidity is estimated from the Helfrich continuum model [38] for bilayer undulations yielding a value of 109 *k*_*b*_*T* at 298 K.

The bending rigidity *k*_*c*_ [37, 38] of a flat bilayer can be obtained from spectral analysis of the fluctuations of the height of the membrane above a reference plane. However, the HIV-1 lipid vesicle is a quasi-spherical membrane that remains intact during the simulation, and therefore the instantaneous shape of the vesicle is decomposed into spherical harmonics [38] (Fig 5).

Following Braun and Sachs [38], the bending rigidity of the viral liposome was obtained from the power spectrum of shape fluctuations in the spherical harmonic basis. The coefficients for the spherical harmonic expansion were obtained from a nonlinear fit of degree 74 and order 149. The undulation power spectrum in Fig 5B and 5C was then computed by fitting *a*_*lm*_ to the power spectrum model in Eq 5, yielding *k*_*c*_ = 109 *k*_*b*_*T* at 298 K. We believe this relatively high bending energy is a result of how we employed the method [38], which required reducing the 5.2 *μ*s timeseries to only 10 frames, with each frame composed of only a subset of headgroups. These measures were taken due to the vesicle’s immense size and lipidomic complexity, which otherwise prevent use of this analysis framework as designed. A framework optimized for large scale, complex lipid systems, founded on the work of Braun and Sachs [38], is in development.

For our asymmetric flat membrane control system (S2 Fig), we employed a method to compute compressibility moduli based on local thickness fluctuations [39, 40], which allows characterization of each leaflet individually. Considering the absence of experimentally-derived reference values gleaned from similar, representative membranes, proper validation of resulting moduli remains a challenge. Nevertheless, the framework [39] is utilized to identify the differences and trends in the compressibility of each leaflet (see Methods for implementation details). For the inner leaflet, we obtained a compressibility modulus of 103 mN/m. For the outer leaflet, we obtained a compressibility modulus of 155 mN/m (S3 Table). Both values were computed at 298 K. Interestingly, our analysis reveals that the outer leaflet, even in the flat patch system where neither leaflet exhibits any sustained curvature, is substantially more rigid than the inner leaflet. Corresponding to our findings related to lateral mobilities, with displacement of lipids in the inner leaflet being markedly higher than the outer leaflet (S10 and S11 Figs), it seems that the asymmetric lipid composition of HIV-1’s membrane leads to significant disparaties in the physical properties of each leaflet.

Flexible envelopes are characteristic of mature HIV-1 virions. Flexibility facilitates entry of mature virions, whereas immature virions exhibit a stiffer viral envelope which cannot enter host cells efficiently [17]. Despite the presence of the low-mobility ordered domains described above, the viral envelope is still clearly in a fluid phase, as the nanoscale ordered domains are transient, and the bending rigidity is still significantly below values reported in the literature for a membrane below the main phase transition temperature [41].

The composition of the outer leaflet of the HIV-1 liposome (rich in sphingolipids and cholesterol) is consistent with raft-like regions of the plasma membrane, from which HIV-1 is believed to escape the cell. In simpler model membrane mixtures, the liquid-ordered phase has several features in common with rafts–it is enriched in high melting temperature lipids (like SM) and cholesterol, the hydrocarbon chains are ordered, and therefore the bending modulus is likely higher than in single component fluid membranes [42, 43]. Recently, Weiner, et al. demonstrated liquid-ordered domain formation in all-atom simulations of more complex mixtures, designed to mimic the asymmetry of the plasma membrane [44].

The transient ordered regions observed in the outer leaflet of the HIV-1 liposome shown in Fig 4 are also observed in bilayer simulations of the liquid-ordered phase [45]. Taken together, the analysis of the HIV-1 liposome simulation indicates that the outer leaflet of the liposome is in an ordered or raft-like phase. The fact that the composition is heterogeneous across the surface of the vesicle suggests that viral assembly might exploit fluctuations in lipid composition to organize surface proteins for critical functions like viral fusion and entry, as suggested previously for influenza [46]. Indeed, Tamm and coworkers have shown that gp41 mediated fusion occurs in heterogeneous membranes at the edge of cholesterol-rich domains [47].

While the absence of gp41 in our simulations precludes any conclusions that may be drawn regarding its effect on the membrane, we have shown that domain formation, potentially requisite for gp41-mediated fusion [47], can occur in protein-free lipid systems. This result suggests that gp41’s hemifusion and fusion activity *in vivo* is contingent upon, rather than causal to, the presence of these cholesterol-rich domains. Interestingly, several studies have shown that removal of cholesterol, or blocking synthesis thereof in virus-producing cells, significantly diminishes HIV-1 infectivity [48, 49]. Because such domains are observed in both the full-scale vesicle as well as the asymmetric flat patch, it is likely that these domains are emergent from sphingolipid and cholesterol-rich lipid mixtures, representative of the sites from where HIV-1 is known to preferentially bud, yet distinct from typical host membrane compositions. Preference toward a specific lipid composition may additionally relate to localization of MA during assembly, further implicating the lipidome in numerous viral processes.

What role is played by proteins in regulating membrane dynamics and behavior is an intriguing question that is made more complicated by the fact that retroviruses are known to freely and unselectively incorporate host membrane proteins into their envelopes [50–53]. The latter fact may be a clue that the physical characteristics of the envelope, those necessary to successful completion of the viral cycle such as stability, fluidity or elasticity, are robust and independent of the action of proteins. Experimental studies of model membranes have shown the presence of gp41’s N-terminal fusion peptide leads to reductions in stiffness [54, 55]. To our knowledge, a similar study with a lipid composition representative of HIV-1, i.e., rich in cholesterol and sphingomyelin, has not been conducted.

## 3 Discussion

We have performed a full-scale simulation of a complex, realistic vesicle at the coarse grain level. The system was built from experimental data, introducing lipid asymmetry in the compositions of the outer and inner leaflet. The results from equilibration show the vesicle maintains sphericity, in agreement with results reported by experimental techniques. In addition, the lipid asymmetry is maintained during the MD production suggesting our model of the vesicle yields an accurate representation of an authentic HIV-1 liposome.

The overall lipid composition of mature HIV-1 virions is known from precision lipidomics data, but compositions specific to each leaflet are not. Although the initial liposome model used in this work is based on existing knowledge of plasma membrane asymmetry [24], it is a hypothesis for the actual lipid distribution. However, during a long, unrestrained production simulation of the full-scale HIV-1 liposome, the initial asymmetry is maintained despite significant occurrences of lipid flip-flop. The latter indicates that the hypothesized distribution is a good approximation of the asymmetric lipid distribution in the viral envelope.

Interestingly, simulations of a flat membrane with the same lipid composition as the full-scale liposome did not include incidents of lipid flip-flop. Aside from further corroborating the studied lipidome as representative of the viral envelope, this result suggests an intimate relationship between curvature, flip-flop and asymmetry. Contrary to the flat membrane, the 150 nm vesicle’s shape is not up/down symmetric, but is curved, with lipids on the outer leaflet under positive curvature, and lipids on the inner leaflet under negative curvature. The distribution of lipids in the HIV-1 liposome appears to match these curvature preferences. Highly unsaturated lipids (especially with small headgroups like PE) prefer negative spontaneous curvatures, while mixtures of sphingomyelin and cholesterol prefer positive spontaneous curvature [56]. Given that HIV-1 buds from specific locations at the plasma membrane and has a lipid composition that is distinct from that of the host cell, this suggests that HIV-1 selects a composition of its envelope to stabilize the virion, by coupling membrane curvature and asymmetric lipid composition.

Further, our lipidomic representation of the HIV-1 vesicle matches the preference of HIV-1 to enrich its bilayer with phosphatidylserine, ceramide, sphingomyelin and cholesterol, as reported empirically for various cell lines [21, 22]. The preferential enrichment of distinct constituents of the lipidome is likely to accommodate morphogenesis, through aforementioned coupling of compositional asymmetry to curvature and to provide domains mimicking detergent-resistant membranes (DRM) with which the Gag polyprotein is known to associate [21].

Viral entry requires the coordinated action of the envelope protein and CD4+ receptors in order to fuse the host cell membrane and the viral envelope. Furthermore, the spatial distribution of the HIV-1 envelope glycoprotein on the surface of the virion is dependent on stage of the life cycle of the virus [57, 58]. Whether lipids play a role in organizing the viral envelope for fusion or during maturation is unknown [57]. However, the observation of fluctuating nanodomains on the (protein-free) viral liposome indicates that this may indeed be the case. These domains are roughly 10 nm in diameter and enriched in sphingolipids, perhaps providing a dynamic platform for the organization of the envelope protein during maturation and fusion.

Further, experimental evidence suggests that the presence of cholesterol in biological membranes leads to entropy driven phase separation of L_*d*_ and L_*o*_ domains [59, 60], where cholesterol participates in either phase without preference to serve the end of hydrophobic tail packing in highly ordered membrane environments. These distinct phases are responsible for heterogeneous lateral diffusion along membrane surfaces. The sphingolipid-enriched, cholesterol-containing domains observed in the present work support this experimental finding. Aside from their potential role in mediating the localization of envelope proteins throughout viral maturation, these domains may serve an additional purpose to aid the packing of lipids within the outer leaflet, where heterogeneous lateral diffusion was observed (Fig 3). Additionally, tight packing of lipids is known to lead to slow lateral diffusion [59], which may explain the distinct difference in lateral mobility observed between the outer and inner leaflets in the HIV-1 liposome, where packing is tighter in the positively curved outer leaflet than in the inner leaflet. This hetereogenous mobility would have an impact on the lateral diffusion of the envelope protein as it would encounter two different lipid environments, namely the outer leaflet by the receptor binding domain of gp41 and the interior leaftlet by its cytoplasmic tail.

Altogether, our study provides insight into the molecular behavior of HIV liposomes with a high level of detail. The lipid vesicle of HIV contains an asymmetric composition of lipids across monolayers, asymmetry which was persistent throughout molecular dynamics simulation. Together with heterogeneous lateral diffusion and the observed transbilayer diffusion, the persistence of macroscopic qualities of the liposome indicates a central role for the vesicle, and its composition, in the HIV viral replication cycle, where such properties are maintained with the intent of stabilizing the virion through maturation, supporting viral protein components and enabling infectivity.

## 4 Methods

### 4.1 Vesicle construction

The approximately 300,000 lipid molecule positions were initially seeded in two spheres, representing leaflets, using the packmol library [61]. The relative abundances of the lipid species were based on the average per-virion values obtained in previous lipidomics experiments for HIV-1 [21, 22] (Table 1). Molecular placement with packmol did not fully converge, so an alchemical phasing procedure was used to progressively expand the effective CG particle radii to relax steric conflicts gradually [62]. A 1,000 frame alchemical growth trajectory spanning λ = 0 to λ = 1 was visually inspected at every 10th frame to determine the point at which lipids had deviated too far from the HIV-1 vesicle because of steric conflicts during lambda-expansion. The acceptable movements were capped at λ = 0.2 (frame 20) and all twenty coordinate sets in that set of frames were energy minimized to assess viability (*F*_*max*_ < 10^4^). The least disruptive (lowest) λ value at which coordinates were viable for energy minimization was frame 7 (λ = 0.08), and these coordinates were selected for preparation of a production simulation.

**Table 1:** Summary of HIV-1 vesicle lipid composition. Lipid names match those used in the MARTINI 2.1 forcefield. There are 24 lipid species in total.

Lipid Name	Number of Molecules
POPS	1,161
DLPC	4,929
PRPS	8,931
DPCE	159
PNSM	10,542
POPC	10,128
POPE	1,092
DOPC	2,855
PQPE	583
PGPS	5,579
PQPS	1,584
DPPC	6,754
PIPE	9,084
PRPE	9,823
CHOL	129,019
PAPE	13,182
PIPS	3,651
DPSM	43,334
PAPS	1,559
PUPE	15,531
DPGS	592
PUPC	776
PRPC	518
PGPE	1,833

The selected lipid vesicle starting configuration was placed in a rhombic dodecahedron container using GROMACS 4.6.5 with a 3.5 nm distance buffer between the vesicle and the simulation container boundary. The dodecahedral system was hydrated with MARTINI water, using a random seed and a van der Waals radius of 0.24 nm. In total, 20,541,842 non-polarizable water particles were added by GROMACS, and the hydrated system was successfully energy-minimized using the steepest descent algorithm in GROMACS. The simulation system was neutralized by replacing 25,000 MARTINI water molecules with Na^+^ ions, followed by another successful steepest descent energy minimization. The final solvent preparation step was to convert to 5% antifreeze particle composition, as described previously [26]. After confirming that the energy minimized solvated coordinates were sensible based on visual inspection, the system was prepared for long-term (production) simulations with GROMACS 2016.

After approximately 1.5 *μ*s of equilibration, we noticed two issues with the HIV-1 vesicle system: there were a few free-floating lipids inside and outside of the ultrastructure, and double bilayer anomalies were present, i.e., regions with more than two lipid leaflets had formed. The bilayer anomalies were excised using a combination of the DBSCAN algorithm [63] in scikit-learn [64] and in-house code to reintegrate false-positive selections.

Following the surgical procedure to remove bilayer anomalies, the vesicular system was rehydrated using GROMACS 4.6.5, with MARTINI water using a van der Waals radius of 0.24 nm. 20,700,856 water particles were added and the hydrated system was energy minimized using the steepest descent algorithm. The hydrated system was neutralized with 22,465 MARTINI sodium ions and again energy minimized with steepest descent. The solvent was converted to 5% antifreeze particles as described above, leaving 19,644,472 conventional waters in the system, and again successfully energy minimized with steepest descent. Following visual inspection of the vesicular system, the extended production simulation was prepared as before using GROMACS 2016.1.

After an additional 1.5 *μ*s of post-surgery vesicle simulation, there was a gap in computational resource availability, and when additional resources were finally secured, we repeated the simulation preparation procedure a third time, this time rehydrating from the final (dehydrated) 1.5 *μ*s post-surgery equilibration snapshot that was still available. 21,666,258 MARTINI waters and 22,465 Na^+^ ions were added, and then solvent was converted to 5% antifreeze particles (1,082,189 added in place of water). Intervening energy minimizations were as described above. To maximize performance, we prepared our long-term production simulation using GROMACS 2018.1.

### 4.2 Simulation parameters

In addition to the lipid components, the system was solvated with 5% antifreeze particles (1,082,189 MARTINI WF residues), 95% water particles (20,561,604 MARTINI W residues), and 22,465 Na^+^ ions, for a total of 24,585,206 beads.

All simulations were performed using the MARTINI 2.1 coarse-grain forcefieldi [30]. After the 1.5 *μ*s initial equilibration using GROMACS [65] version 2016.4, 5.2 *μ*s production simulations were performed using GROMACS 2018.1 with 10 fs timesteps, the Verlet cutoff scheme, and reaction field electrostatics. Isotropic pressure coupling was employed using the Berendsen barostat at a time constant of 20 ps, compressibility of 1 × 10^−6^ bar^−1^, and reference pressure of 1.0 bar. Lipids and solvent were separately temperature coupled with a 323 K reference temperature using the Berendsen thermostat, and a 1 ps time constant.

### 4.3 Asymmetric flat membrane

To construct the 46 × 46 nm, asymmetric flat membrane patch, we first used CHARMM-GUI’s Martini Maker tool [66] to create two, symmetric bilayers representing the compositions of the inner and outer leaflets of the HIV-1 liposome. Employing the same CG MARTINI lipid, water and ion models as the vesicle system, each symmetric bilayer was minimized, thermalized then equilibrated for 500 ns using GROMACS 2021.6. With the equilibrated bilayers, we then divided and merged the two systems to create a new asymmetric bilayer, preserving water and ions, and thus the hydrophilic interactions, associated with each equilibrated leaflet. Following the merging procedure, VMD’s atomselect feature was employed to trim overhanging lipids in the X and Y dimensions from the slightly-larger inner leaflet.

The composite system of merged leaflets was then minimized and thermalized. Headgroup restraints were employed with a force constant of 20 kJ/mol, and these were gradually relaxed over the course of 1 *μ*s to allow tail melting. Once restraints were released completely and the unrestrained system was allowed to equilibrate for 1 *μ*s, we simulated the asymmetric flat patch for a total of 7 *μ*s of production sampling. Simulation parameters were chosen to match those described in the previous section for the HIV-1 vesicle system.

### 4.4 Analysis of simulations

Trajectory data files were read and exposed to the Python interpreter using the MDAnalysis library [67, 68]. Trajectories were visualized using VMD [69], and analysis plots were produced using matplotlib [70]. Array-based calculations of simulation properties leveraged the NumPy library [71]. The SciPy library [72] was used for a number of scientific algorithms. Average mean and Gaussian curvature analyses of the asymmetric flat patch were accomplished using the MDAnalysis library [67, 68].

#### 4.4.1 Sphericity tracking

Sphericity of the liposome was calculated using the ratio between surface area and volume previously reported in a geological context [73]. The surface area of the vesicle was estimated by recasting the Cartesian coordinates of the headgroups to spherical coordinates and taking the average of the radius of the system centered at the origin. Based on the sphericity tracking analysis during equilibration, the surface area was estimated for the circumference encompassing the average radius of the headgroups of both monolayers.

### 4.5 Hydrophobic thickness

Hydrophobic thickness was considered as the center of mass distance between uniformly distributed groups of the first acyl carbon bead (‘C2B’) in each leaflet. Leaflets were delineated via VMD *measure volinterior* [32], and groups of beads were considered in a radius of 46.25 Å from *N* = 1024 test points, the latter uniformly distributed via a Poisson disk sampling procedure written by the authors [32].

### 4.6 Lipid translational and trans-bilayer diffusion

The translational diffusion coefficients of the lipids in each leaflet were calculated using the approach previously reported for viral simulations [27] using,

$$\begin{array}{c} MSD=4Dt^{\alpha }. \end{array}$$ (1)

Where MSD represents the mean square displacement (MSD) of the centroid of every lipid, *D* is the lateral diffusion coefficient and *α* is an arbitrary scaling factor. *D* and *α* were estimated using linear least-square fitting of MSD for simulation windows of size: 5, 25, 50, 100, 250, 500, 1000 ns. The standard deviations for the diffusion coefficient and the scaling factor were derived from the square root of the covariance matrix of the linear least-square fitting as previously described [27].

Analysis of trans-bilayer diffusion measured the number of flip-flop events per unit of time, for each lipid species, using a 3D space classifier developed by the authors [32]. Using lipid tail groups, the volume of the vesicle system was classified such that molecular density corresponding to the tail group selection served as a barrier separating interior and exterior regions of the system, representing the inner and outer leaflets, respectively. For each frame of the trajectory, headgroups were characterized according to their positions within the classified volume, i.e., interior or exterior. The latter was made possible through a massively parallel analysis framework [29]. To mitigate error resulting from undulations of the vesicle, volume classification was repeated every 20 ns throughout the time series analysis. Data resulting from headgroup tracking were used to quantify inner-to-outer and outer-to-inner translocation events and determine effective rates thereof for each species.

### 4.7 Mobility analysis

Lateral mobility of lipids was measured by estimating the root mean square fluctuation (RMSF) as $RMSF_{\text{ith}\;\text{headgroup}}=\langle (r_{i}-\langle r_{i}\rangle {)}^{2}\rangle^{\frac{1}{2}}$; where $\langle x\rangle =\frac{1}{N_{\text{frames}}}\sum_{\text{n}=\text{frames}}\;x_{n}$. RMSF values were calculated for lipid headgroups over a simulation window of size of 1 *μ*s. Results are reported as the mean RMSF for headgroups of each lipid in the vesicle.

### 4.8 Bending rigidity

The bending rigidity of the full-scale vesicle was calculated from a well-established spherical harmonic analysis for spherical liposomes [38]. First, a single reference frame is defined by estimating the undulating radial surface (URS) [38]. The initial URS is estimated by recasting a *ψ*,*θ*-grid from the coordinate system of the vesicle, where *θ* ∈ [0, *ψ*] and *ψ* ∈ [0, 2*π*], for the colatitude and longitude, respectively. A total of two inital grids were generated, each one corresponding to one monolayer of the vesicle. The angular resolution for the two grids was *dθ* = *dψ* = 0.042 rad. based on a cutoff wavenumber of 0.5 nm^-1^ and a cutoff filter of 2.5 nm^-1^ to mitigate discontinuities at the poles of the vesicle [38]. After defining the surface of the inner (*r*_*in*_(*ϕ*, *ψ*)) and outer monolayer (*r*_*out*_(*ϕ*, *ψ*)), the undulating surface *r*_*und*_ was calculated for the vesicle as shown in Eq 2.

$$\begin{array}{c} r_{und}=\frac{1}{2}(r_{out}\left(\theta ,\phi \right)+r_{in}\left(\theta ,\phi \right)) \end{array}$$ (2)

The average radius of the undulating surface $r_{0}^{\prime }$ was then used to define the normalized radial fluctuation as

$$\begin{array}{c} f\left(\theta ,\phi \right)=\frac{r_{und}\left(\theta ,\phi \right)-r_{0}^{\prime }}{r_{0}^{\prime }} \end{array}$$ (3)

Subsequently, spherical harmonic analysis was employed to decompose the normalized radial fluctuations from Eq 3. Liposome fluctuations were expanded in spherical harmonics of degree *l* and order *m* as

$$\begin{array}{c} f\left(\theta ,\phi \right)=\sum_{l,m}a_{l,m}Y_{l,m} \end{array}$$ (4)

The linear combination of spherical harmonics from Eq 4 consists of harmonic coefficients *a*_*l*,*m*_ and basis functions $Y_{l,m}=P_{m}^{l}\;\text{cos}\;\theta e^{i\phi }$; where $P_{m}^{l}$ are Legendre polynomials. The order and degree of *Y*_*l*,*m*_ are dependent on the number of points describing the initial undulating surface. In the case of the HIV-1 vesicle, the spherical harmonics were expanded as a linear combination of degree 74 and order 149.

Finally, the pseudo-inverse of the matrix *Y* composed by the basis functions is used to estimate the harmonic coefficients *a*_*l*,*m*_. The *a*_*lm*_ can be used to obtain the power spectra of undulations by fitting the harmonic coefficients to the Helfrich continuum model for undulations on a sphere with vanishing spontaneous curvature [74], shown in Eq 5. The fitting yields an estimation of the bending rigidity *k*_*c*_ at temperature *T* as

$$\begin{array}{c} |a_{l,m}{|}^{2}=\frac{k_{b}T}{k_{c}[l^{2}{\left(l+1\right)}^{2}-2l\left(l+1\right)]}, \end{array}$$ (5)

where *k*_*b*_ is the Boltzmann constant.

### 4.9 Compressibility analysis

We analyzed compressibility using a technique outlined by Doktorova et al. [39], which enables the calculation of individual moduli for each leaflet in the asymmetric system. In particular, we followed the approach based on fluctuations in local thickness. To render the coarse, x-y interpolated grids from which local fluctuations are measured, we used ‘PO4’, ‘C2B’ and ‘C4B’ beads within each leaflet. Thoughtful consideration toward selection of atoms or beads used in defining these surfaces is given by the authors in the method’s paper [39].

Following guidance outlined by the authors, we chose an interpolation radius of 4.6 nm in the x-y plane, which is slightly larger than the bilayer’s overall thickness. This radius was used to average positions of the three selected beads within their respective grid cells. The analysis was accomplished with in-house Tcl procedures, invoked within VMD. Kernel density estimation, for determinations of equilibrium area and thickness used in computing compressibility, was accomplished using the Tcl8.5 ‘::math::statistics’ package. It should be noted that moduli obtained from fluctuations in lateral area, another approach outlined by Doktorova et al., is unsuitable for our flat membrane systems because their large areas lead to underestimations of mechanical stiffness [39].

## Supporting information

S1 Fig Chemical structures of lipid species comprising the HIV-1 lipidome. (TIF)

S2 Fig Asymmetric, flat HIV-1 membrane. (TIF)

S3 Fig Outer diameter and sphericity analysis of the HIV-1 vesicle, equilibration. (TIF)

S4 Fig Outer diameter and sphericity analysis of the HIV-1 vesicle, production. (TIF)

S5 Fig Lipid flip-flop, transverse diffusion, rates for the HIV-1 vesicle. (TIF)

S6 Fig Linear fit of the mean square displacement (MSD) analysis. (TIF)

S7 Fig Composition of the vesicle according to distribution of bilayer thickness values. (TIF)

S8 Fig Lipid composition of the authentic HIV-1 liposome. (TIF)

S9 Fig Visualization of the HIV-1 MARTINI lipidome. (TIF)

S10 Fig Distribution of lipid headgroup mobility and Gaussian fit. (TIF)

S11 Fig Mean square displacement (MSD) profiles for asymmetric, flat HIV-1 membrane. (TIF)

S1 Table Lipidome of the HIV-1 vesicle. (PDF)

S2 Table Chemical names and corresponding MARTINI residue names for the HIV-1 lipidome. (PDF)

S3 Table Compressibility moduli for flat, HIV-1 membrane systems. (PDF)

S1 Movie Molecular dynamics simulation of the full-scale HIV-1 vesicle. (MP4)

S2 Movie Visualization of lipid flip-flop from HIV-1 vesicle simulation. (MP4)

S3 Movie High and low mobility regions of the HIV-1 vesicle. (MP4)

## References

[1] Murin CD, Wilson IA, Ward AB. Antibody responses to viral infections: a structural perspective across three different enveloped viruses. Nature microbiology. 2019;4(5):734–747. doi: 10.1038/s41564-019-0392-y 30886356 PMC6818971

[2] Chazal N, Gerlier D. Virus entry, assembly, budding, and membrane rafts. Microbiology and molecular biology reviews. 2003;67(2):226–237. doi: 10.1128/MMBR.67.2.226-237.2003 12794191 PMC156468

[3] Zhang Z, He G, Filipowicz NA, Randall G, Belov GA, Kopek BG, et al. Host lipids in positive-strand RNA virus genome replication. Frontiers in microbiology. 2019;10:286. doi: 10.3389/fmicb.2019.00286 30863375 PMC6399474

[4] Kerviel A, Thomas A, Chaloin L, Favard C, Muriaux D. Virus assembly and plasma membrane domains: which came first? Virus research. 2013;171(2):332–340. doi: 10.1016/j.virusres.2012.08.014 22989508

[5] Ono A, Freed EO. Plasma membrane rafts play a critical role in HIV-1 assembly and release. Proceedings of the National Academy of Sciences. 2001;98(24):13925–13930. doi: 10.1073/pnas.241320298 11717449 PMC61143

[6] Ono A, Ablan SD, Lockett SJ, Nagashima K, Freed EO. Phosphatidylinositol (4, 5) bisphosphate regulates HIV-1 Gag targeting to the plasma membrane. Proceedings of the National Academy of Sciences. 2004;101(41):14889–14894. doi: 10.1073/pnas.0405596101 15465916 PMC522033

[7] Murphy RE, Saad JS. The Interplay between HIV-1 Gag Binding to the Plasma Membrane and Env Incorporation. Viruses. 2020;12(5):548. doi: 10.3390/v12050548 PMC7291237 32429351

[8] Campbell EM, Hope TJ. HIV-1 capsid: the multifaceted key player in HIV-1 infection. Nature Reviews Microbiology. 2015;13(8):471–483. doi: 10.1038/nrmicro3503 26179359 PMC4876022

[9] Freed EO. HIV-1 assembly, release and maturation. Nature Reviews Microbiology. 2015;13(8):484–496. doi: 10.1038/nrmicro3490 26119571 PMC6936268

[10] Harrison SC. Viral membrane fusion. Nature structural & molecular biology. 2008;15(7):690–698. doi: 10.1038/nsmb.1456 18596815 PMC2517140

[11] Harrison SC. Mechanism of membrane fusion by viral envelope proteins. Advances in virus research. 2005;64:231–261. doi: 10.1016/S0065-3527(05)64007-9 16139596 PMC7173036

[12] Sengupta P, Seo AY, Pasolli HA, Song YE, Johnson MC, Lippincott-Schwartz J. A lipid-based partitioning mechanism for selective incorporation of proteins into membranes of HIV particles. Nature cell biology. 2019;21(4):452–461. doi: 10.1038/s41556-019-0300-y 30936472

[13] Mücksch F, Laketa V, Müller B, Schultz C, Kräusslich HG. Synchronized HIV assembly by tunable PIP2 changes reveals PIP2 requirement for stable Gag anchoring. Elife. 2017;6:e25287. doi: 10.7554/eLife.25287 28574338 PMC5495570

[14] Wen Y, Feigenson GW, Vogt VM, Dick RA. Mechanisms of PI (4, 5) P2 Enrichment in HIV-1 Viral Membranes. Journal of Molecular Biology. 2020;432(19):5343–5364. doi: 10.1016/j.jmb.2020.07.018 32739462 PMC8262684

[15] Monje-Galvan V, Voth GA. Binding mechanism of the matrix domain of HIV-1 gag on lipid membranes. Elife. 2020;9:e58621. doi: 10.7554/eLife.58621 32808928 PMC7476761

[16] McMahon HT, Gallop JL. Membrane curvature and mechanisms of dynamic cell membrane remodelling. Nature. 2005;438(7068):590–596. doi: 10.1038/nature04396 16319878

[17] Kol N, Shi Y, Tsvitov M, Barlam D, Shneck RZ, Kay MS, et al. A stiffness switch in human immunodeficiency virus. Biophysical journal. 2007;92(5):1777–1783. doi: 10.1529/biophysj.106.093914 17158573 PMC1796819

[18] Pang HB, Hevroni L, Kol N, Eckert DM, Tsvitov M, Kay MS, et al. Virion stiffness regulates immature HIV-1 entry. Retrovirology. 2013;10(1):4. doi: 10.1186/1742-4690-10-4 23305456 PMC3564805

[19] Trautz B, Wiedemann H, Lüchtenborg C, Pierini V, Kranich J, Glass B, et al. The host-cell restriction factor SERINC5 restricts HIV-1 infectivity without altering the lipid composition and organization of viral particles. Journal of Biological Chemistry. 2017;292(33):13702–13713. doi: 10.1074/jbc.M117.797332 28659343 PMC5566525

[20] Floderer C, Masson JB, Boilley E, Georgeault S, Merida P, El Beheiry M, et al. Single molecule localisation microscopy reveals how HIV-1 Gag proteins sense membrane virus assembly sites in living host CD4 T cells. Scientific reports. 2018;8(1):1–15. doi: 10.1038/s41598-018-34536-y 30389967 PMC6214999

[21] Brugger B, Glass B, Haberkant P, Leibrecht I, Wieland FT, Krausslich HG. The HIV lipidome: a raft with an unusual composition. Proc Natl Acad Sci USA. 2006;103(8):2641–2646. doi: 10.1073/pnas.0511136103 16481622 PMC1413831

[22] Lorizate M, Sachsenheimer T, Glass B, Habermann A, Gerl MJ, Kräusslich HG, et al. Comparative lipidomics analysis of HIV-1 particles and their producer cell membrane in different cell lines. Cellular microbiology. 2013;15(2):292–304. doi: 10.1111/cmi.12101 23279151

[23] Van Meer G, Voelker DR, Feigenson GW. Membrane Lipids: Where They are and How They Behave. Nature reviews Molecular cell biology. 2008;9(2):112–124. doi: 10.1038/nrm2330 18216768 PMC2642958

[24] Lorent J, Levental K, Ganesan L, Rivera-Longsworth G, Sezgin E, Doktorova M, et al. Plasma membranes are asymmetric in lipid unsaturation, packing and protein shape. Nature Chemical Biology. 2020;16(6):644–652. doi: 10.1038/s41589-020-0529-6 32367017 PMC7246138

[25] Perilla JR, Goh BC, Cassidy CK, Liu B, Bernardi RC, Rudack T, et al. Molecular dynamics simulations of large macromolecular complexes. Curr Opin Struct Biol. 2015;31:64–74. doi: 10.1016/j.sbi.2015.03.007 25845770 PMC4476923

[26] Reddy T, Shorthouse D, Parton DL, Jefferys E, Fowler PW, Chavent M, et al. Nothing to sneeze at: a dynamic and integrative computational model of an influenza A virion. Structure. 2015;23(3):584–597. doi: 10.1016/j.str.2014.12.019 25703376 PMC4353694

[27] Reddy T, Sansom MS. The Role of the Membrane in the Structure and Biophysical Robustness of the Dengue Virion Envelope. Structure. 2016;24(3):375–382. doi: 10.1016/j.str.2015.12.011 26833387 PMC4780862

[28] Marrink SJ, Corradi V, Souza PC, Ingólfsson HI, Tieleman DP, Sansom MS. Computational modeling of realistic cell membranes. Chemical reviews. 2019;119(9):6184–6226. doi: 10.1021/acs.chemrev.8b00460 30623647 PMC6509646

[29] Gonzalez-Arias F, Reddy T, Stone JE, Hadden-Perilla JA, Perilla JR. Scalable Analysis of Authentic Viral Envelopes on FRONTERA. Computing in Science & Engineering. 2020;22(6):11–20. doi: 10.1109/mcse.2020.3020508 33510584 PMC7839976

[30] Monticelli L, Kandasamy SK, Periole X, Larson RG, Tieleman DP, Marrink SJ. The MARTINI Coarse-Grained Force Field: Extension to Proteins. J Chem Theory Comput. 2008;4(5):819–834. doi: 10.1021/ct700324x 26621095

[31] Benjamin J, Ganser-Pornillos BK, Tivol WF, Sundquist WI, Jensen GJ. Three-dimensional structure of HIV-1 virus-like particles by electron cryotomography. Journal of molecular biology. 2005;346(2):577–588. doi: 10.1016/j.jmb.2004.11.064 15670606 PMC6608732

[32] Bryer AJ, Hadden-Perilla JA, Stone JE, Perilla JR. High-performance analysis of biomolecular containers to measure small-molecule transport, transbilayer lipid diffusion, and protein cavities. Journal of chemical information and modeling. 2019;59(10):4328–4338. doi: 10.1021/acs.jcim.9b00324 31525965 PMC6817393

[33] Cornell CE, Mileant A, Thakkar N, Lee KK, Keller SL. Direct imaging of liquid domains in membranes by cryo-electron tomography. Proceedings of the National Academy of Sciences. 2020;117(33):19713–19719. doi: 10.1073/pnas.2002245117 32759217 PMC7443872

[34] Gu RX, Baoukina S, Tieleman DP. Cholesterol flip-flop in heterogeneous membranes. Journal of chemical theory and computation. 2019;15(3):2064–2070. doi: 10.1021/acs.jctc.8b00933 30633868

[35] Waheed AA, Freed EO. Lipids and membrane microdomains in HIV-1 replication. Virus research. 2009;143(2):162–176. doi: 10.1016/j.virusres.2009.04.007 19383519 PMC2731011

[36] Guyader M, Kiyokawa E, Abrami L, Turelli P, Trono D. Role for human immunodeficiency virus type 1 membrane cholesterol in viral internalization. Journal of virology. 2002;76(20):10356–10364. doi: 10.1128/JVI.76.20.10356-10364.2002 12239312 PMC136590

[37] Tarazona P, Chacón E, Bresme F. Thermal fluctuations and bending rigidity of bilayer membranes. The Journal of chemical physics. 2013;139(9):094902. doi: 10.1063/1.4818421 24028128

[38] Braun AR, Sachs JN. Determining structural and mechanical properties from molecular dynamics simulations of lipid vesicles. Journal of chemical theory and computation. 2014;10(9):4160–4168. doi: 10.1021/ct500460u 25221448 PMC4159217

[39] Doktorova M, LeVine MV, Khelashvili G, Weinstein H. A new computational method for membrane compressibility: Bilayer mechanical thickness revisited. Biophysical journal. 2019;116(3):487–502. doi: 10.1016/j.bpj.2018.12.016 30665693 PMC6369663

[40] Nagle JF. Area compressibility moduli of the monolayer leaflets of asymmetric bilayers from simulations. Biophysical journal. 2019;117(6):1051–1056. doi: 10.1016/j.bpj.2019.08.016 31493860 PMC6818143

[41] Dimova R, Pouligny B, Dietrich C. Pretransitional effects in dimyristoylphosphatidylcholine vesicle membranes: optical dynamometry study. Biophysical Journal. 2000;79(1):340–356. doi: 10.1016/S0006-3495(00)76296-5 10866960 PMC1300938

[42] Levental I, Levental KR, Heberle FA. Lipid rafts: controversies resolved, mysteries remain. Trends in cell biology. 2020. doi: 10.1016/j.tcb.2020.01.009 32302547 PMC7798360

[43] Levental I. Lipid rafts come of age. Nature Reviews Molecular Cell Biology. 2020; p. 420–420. doi: 10.1038/s41580-020-0252-x 32350456

[44] Weiner MD, Feigenson GW. Molecular dynamics simulations reveal leaflet coupling in compositionally asymmetric phase-separated lipid membranes. The Journal of Physical Chemistry B. 2019;123(18):3968–3975. doi: 10.1021/acs.jpcb.9b03488 31009218

[45] Sodt AJ, Sandar ML, Gawrisch K, Pastor RW, Lyman E. The molecular structure of the liquid-ordered phase of lipid bilayers. Journal of the American Chemical Society. 2014;136(2):725–732. doi: 10.1021/ja4105667 24345334 PMC4197129

[46] Goronzy I, Rawle R, Boxer S, Kasson PM. Cholesterol enhances influenza binding avidity by controlling nanoscale receptor clustering. Chemical science. 2018;9(8):2340–2347. doi: 10.1039/c7sc03236f 29520318 PMC5839467

[47] Yang ST, Kiessling V, Simmons JA, White JM, Tamm LK. HIV gp41–mediated membrane fusion occurs at edges of cholesterol-rich lipid domains. Nature chemical biology. 2015;11(6):424. doi: 10.1038/nchembio.1800 25915200 PMC4433777

[48] Chen B. Molecular mechanism of HIV-1 entry. Trends in microbiology. 2019;27(10):878–891. doi: 10.1016/j.tim.2019.06.002 31262533 PMC6744290

[49] Ono A, Waheed AA, Freed EO. Depletion of cellular cholesterol inhibits membrane binding and higher-order multimerization of human immunodeficiency virus type 1 Gag. Virology. 2007;360(1):27–35. doi: 10.1016/j.virol.2006.10.011 17095032 PMC1945131

[50] Checkley MA, Luttge BG, Freed EO. HIV-1 envelope glycoprotein biosynthesis, trafficking, and incorporation. Journal of molecular biology. 2011;410(4):582–608. doi: 10.1016/j.jmb.2011.04.042 21762802 PMC3139147

[51] Lusso P, di Marzo Veronese F, Ensoli B, Franchini G, Jemma C, DeRocco SE, et al. Expanded HIV-1 cellular tropism by phenotypic mixing with murine endogenous retroviruses. Science. 1990;247(4944):848–852. doi: 10.1126/science.2305256 2305256

[52] Arthur LO, Bess J, Sowder RC, Benveniste RE, Mann DL, Chermann JC, et al. Cellular proteins bound to immunodeficiency viruses: implications for pathogenesis and vaccines. Science. 1992;258(5090):1935–1938. doi: 10.1126/science.1470916 1470916

[53] Ott DE. Cellular proteins detected in HIV-1. Reviews in medical virology. 2008;18(3):159–175. doi: 10.1002/rmv.570 18265424

[54] Shchelokovskyy P, Tristram-Nagle S, Dimova R. Effect of the HIV-1 fusion peptide on the mechanical properties and leaflet coupling of lipid bilayers. New journal of physics. 2011;13(2):025004. doi: 10.1088/1367-2630/13/2/025004 23505334 PMC3595596

[55] Tristram-Nagle S, Nagle JF. HIV-1 fusion peptide decreases bending energy and promotes curved fusion intermediates. Biophysical journal. 2007;93(6):2048–2055. doi: 10.1529/biophysj.107.109181 17526585 PMC1959562

[56] Sodt A, Venable R, Lyman E, Pastor R. Nonadditive compositional curvature energetics of lipid bilayers. Physical review letters. 2016;117(13):138104. doi: 10.1103/PhysRevLett.117.138104 27715135 PMC5134905

[57] Chojnacki J, Waithe D, Carravilla P, Huarte N, Galiani S, Enderlein J, et al. Envelope glycoprotein mobility on HIV-1 particles depends on the virus maturation state. Nature communications. 2017;8(1):1–10. doi: 10.1038/s41467-017-00515-6 28916807 PMC5601426

[58] Wang Q, Finzi A, Sodroski J. The Conformational States of the HIV-1 Envelope Glycoproteins. Trends in Microbiology. 2020. doi: 10.1016/j.tim.2020.03.007 32418859 PMC7363548

[59] Lindblom G, Orädd G. Lipid lateral diffusion and membrane heterogeneity. Biochimica et Biophysica Acta (BBA)-Biomembranes. 2009;1788(1):234–244. doi: 10.1016/j.bbamem.2008.08.016 18805393

[60] Jeon JH, Monne HMS, Javanainen M, Metzler R. Anomalous diffusion of phospholipids and cholesterols in a lipid bilayer and its origins. Physical review letters. 2012;109(18):188103. doi: 10.1103/PhysRevLett.109.188103 23215336

[61] Martinez L, Andrade R, Birgin EG, Martinez JM. PACKMOL: a package for building initial configurations for molecular dynamics simulations. J Comput Chem. 2009;30(13):2157–2164. doi: 10.1002/jcc.21224 19229944

[62] Jefferys E, Sands ZA, Shi J, Sansom MS, Fowler PW. Alchembed: A Computational Method for Incorporating Multiple Proteins into Complex Lipid Geometries. J Chem Theory Comput. 2015;11(6):2743–2754. doi: 10.1021/ct501111d 26089745 PMC4467903

[63] Ester M, Kriegel HP, Sander J, Xu X. A Density-Based Algorithm for Discovering Clusters a Density-Based Algorithm for Discovering Clusters in Large Spatial Databases with Noise. KDD’96. AAAI Press; 1996.

[64] Pedregosa F, Varoquaux G, Gramfort A, Michel V, Thirion B, Grisel O, et al. Scikit-learn: Machine Learning in Python. Journal of Machine Learning Research. 2011;12:2825–2830.

[65] Abraham MJ, Murtola T, Schulz R, Páll S, Smith JC, Hess B, et al. GROMACS: High Performance Molecular Simulations Through Multi-level Parallelism From Laptops to Supercomputers. SoftwareX. 2015;1-2:19–25. doi: 10.1016/j.softx.2015.06.001

[66] Qi Y, Ingólfsson HI, Cheng X, Lee J, Marrink SJ, Im W. CHARMM-GUI martini maker for coarse-grained simulations with the martini force field. Journal of chemical theory and computation. 2015;11(9):4486–4494. doi: 10.1021/acs.jctc.5b00513 26575938

[67] Richard J Gowers, Max Linke, Jonathan Barnoud, Tyler J E Reddy, Manuel N Melo, Sean L Seyler, et al. MDAnalysis: A Python Package for the Rapid Analysis of Molecular Dynamics Simulations. In: Sebastian Benthall, Scott Rostrup, editors. Proceedings of the 15th Python in Science Conference; 2016. p. 98–105.

[68] Michaud-Agrawal N, Denning EJ, Woolf TB, Beckstein O. MDAnalysis: A Toolkit for the Analysis of Molecular Dynamics Simulations. Journal of Computational Chemistry. 2011;32(10):2319–2327. doi: 10.1002/jcc.21787 21500218 PMC3144279

[69] Humphrey W, Dalke A, Schulten K. VMD—Visual Molecular Dynamics. Journal of Molecular Graphics. 1996;14:33–38. doi: 10.1016/0263-7855(96)00018-5 8744570

[70] Hunter JD. Matplotlib: A 2D graphics environment. Computing in Science & Engineering. 2007;9(3):90–95. doi: 10.1109/MCSE.2007.55

[71] Harris C.R., Millman K.J., van der Walt S.J. et al. Array programming with NumPy. Nature 585, 357–362 (2020). doi: 10.1038/s41586-020-2649-2 32939066 PMC7759461

[72] Virtanen P, Gommers R, Oliphant TE, Haberland M, Reddy T, Cournapeau D, et al. (2020) SciPy 1.0–Fundamental Algorithms for Scientific Computing in Python. Nature Methods, 17(3), 261–272. 32015543 10.1038/s41592-019-0686-2 PMC7056644

[73] Wadell H. Volume, Shape, and Roundness of Quartz Particles. Journal of Geology. 1935;43(3):250–280. doi: 10.1086/624298

[74] Helfrich W. Size distributions of vesicles: the role of the effective rigidity of membranes. Journal de physique. 1986;47(2):321–329. doi: 10.1051/jphys:01986004702032100

