# Supplementary figures and images for: Full scale structural, mechanical and dynamical properties of HIV-1 liposomes
Source: PLoS Comput Biol. 2022 Jan 18;18(1):e1009781. doi: 10.1371/journal.pcbi.1009781 (PMC8797243; doi:10.1371/journal.pcbi.1009781)

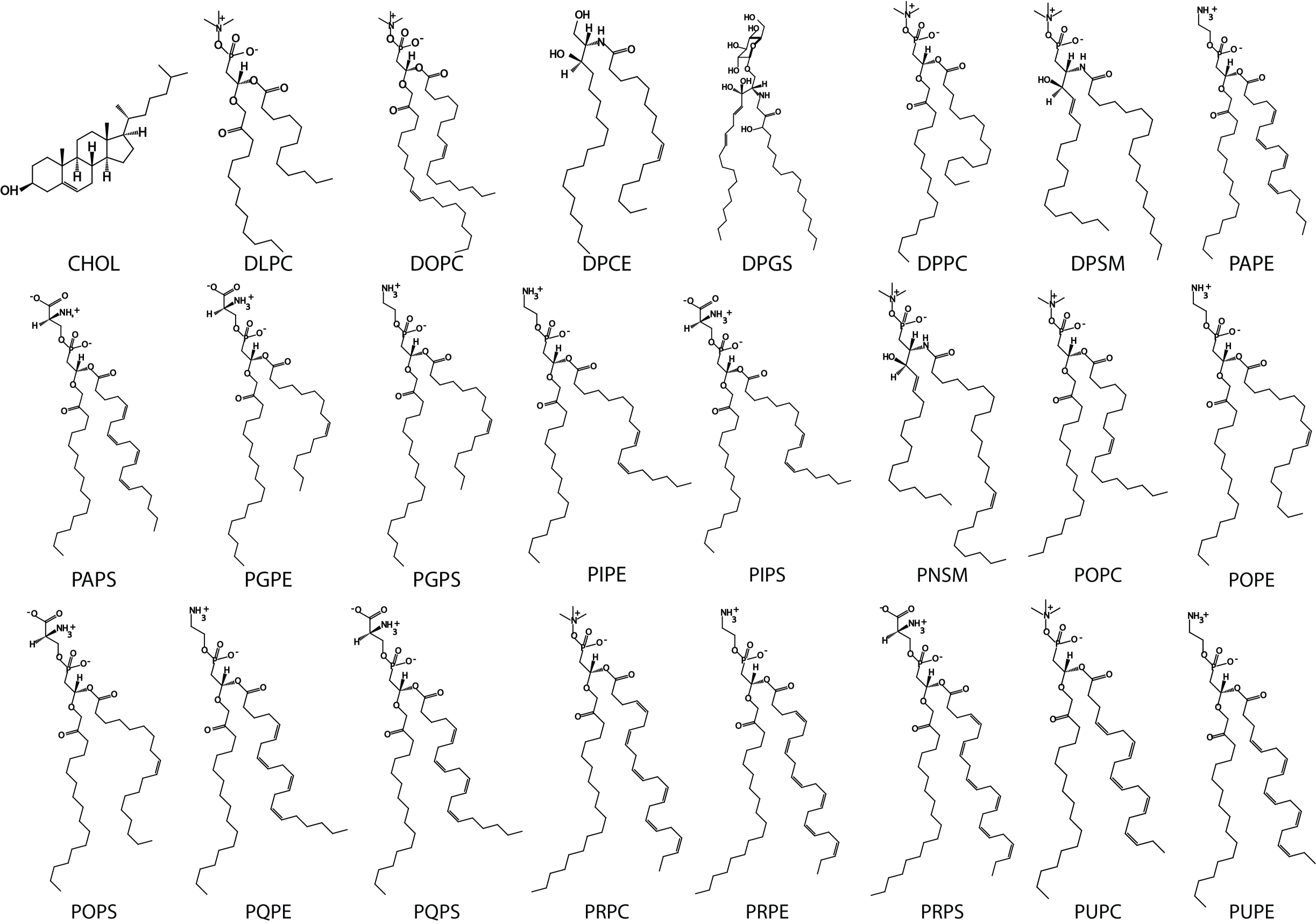

Supplement: S1 Fig — (TIF) [file pcbi.1009781.s001.tif]

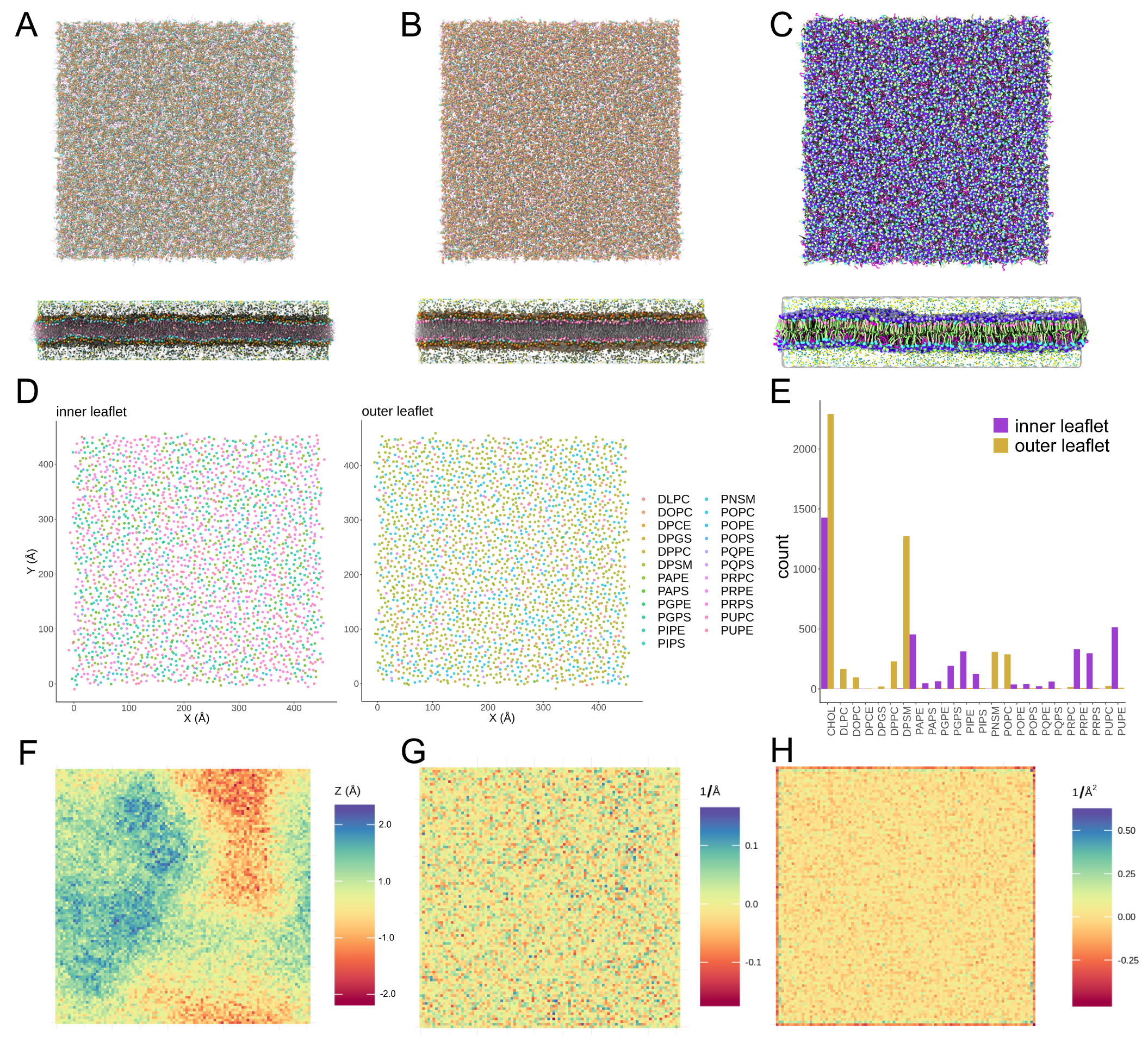

Supplement: S2 Fig — (TIF) [file pcbi.1009781.s002.tif]

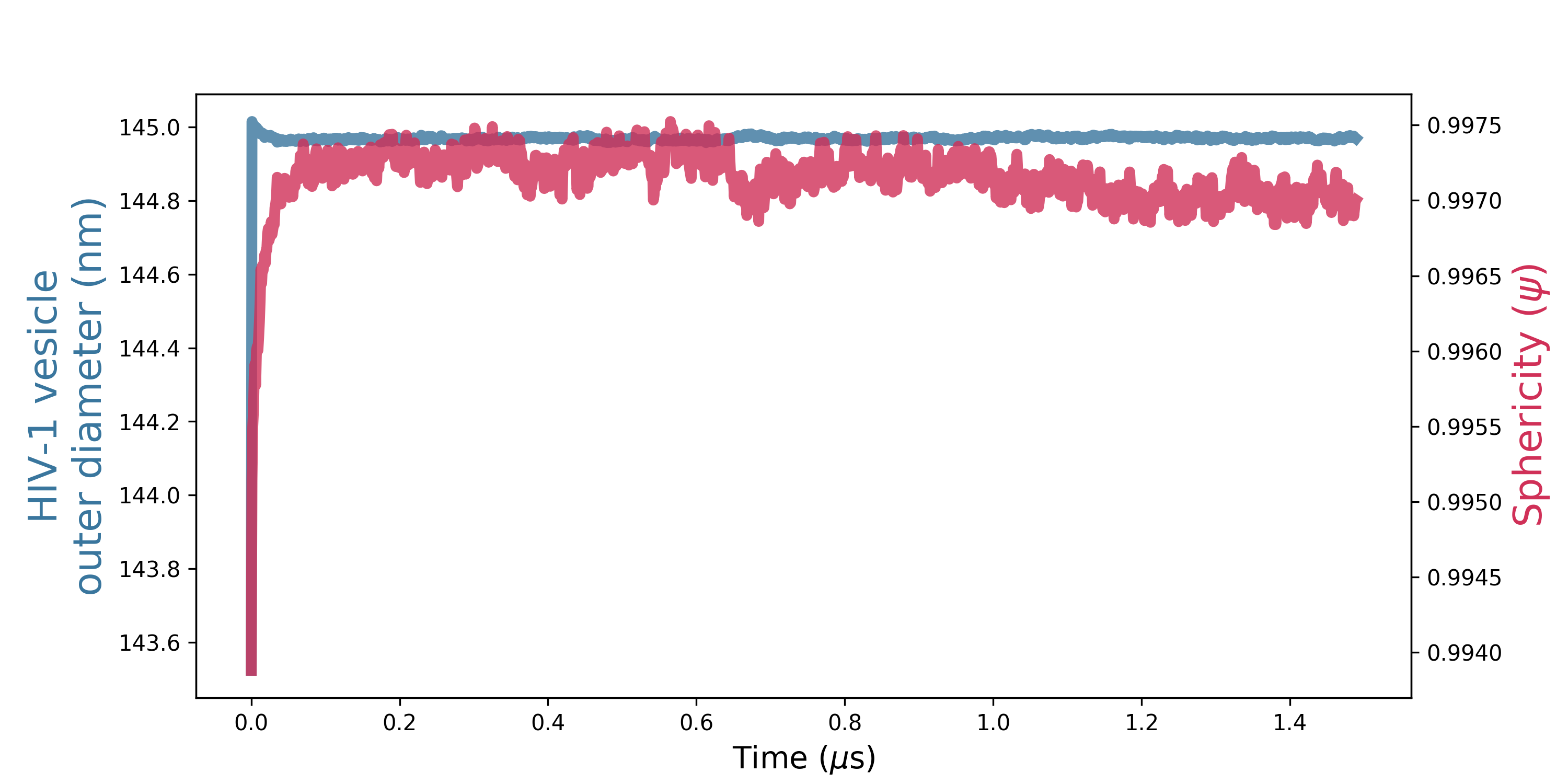

Supplement: S3 Fig — (TIF) [file pcbi.1009781.s003.tif]

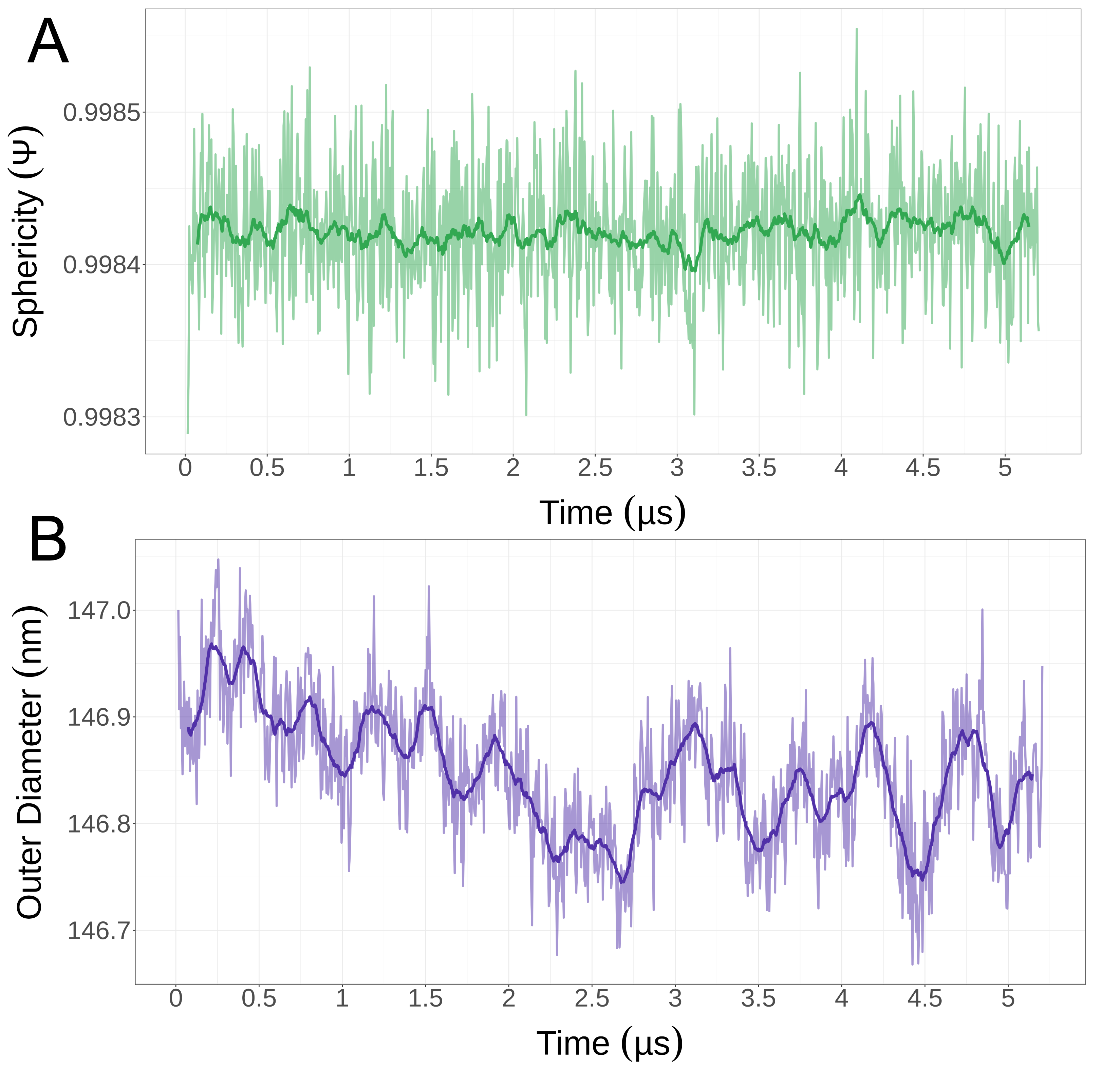

Supplement: S4 Fig — (TIF) [file pcbi.1009781.s004.tif]

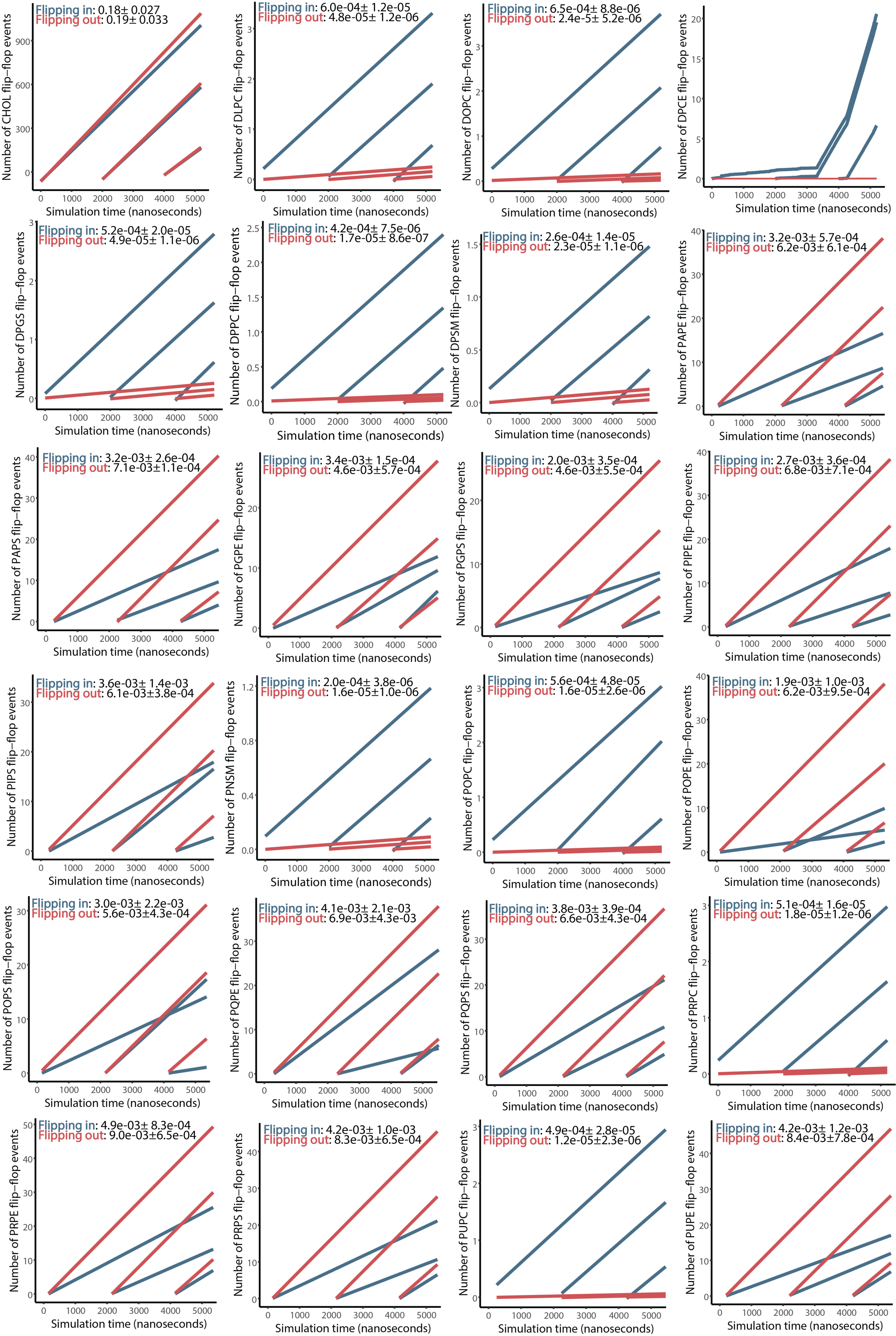

Supplement: S5 Fig — (TIF) [file pcbi.1009781.s005.tif]

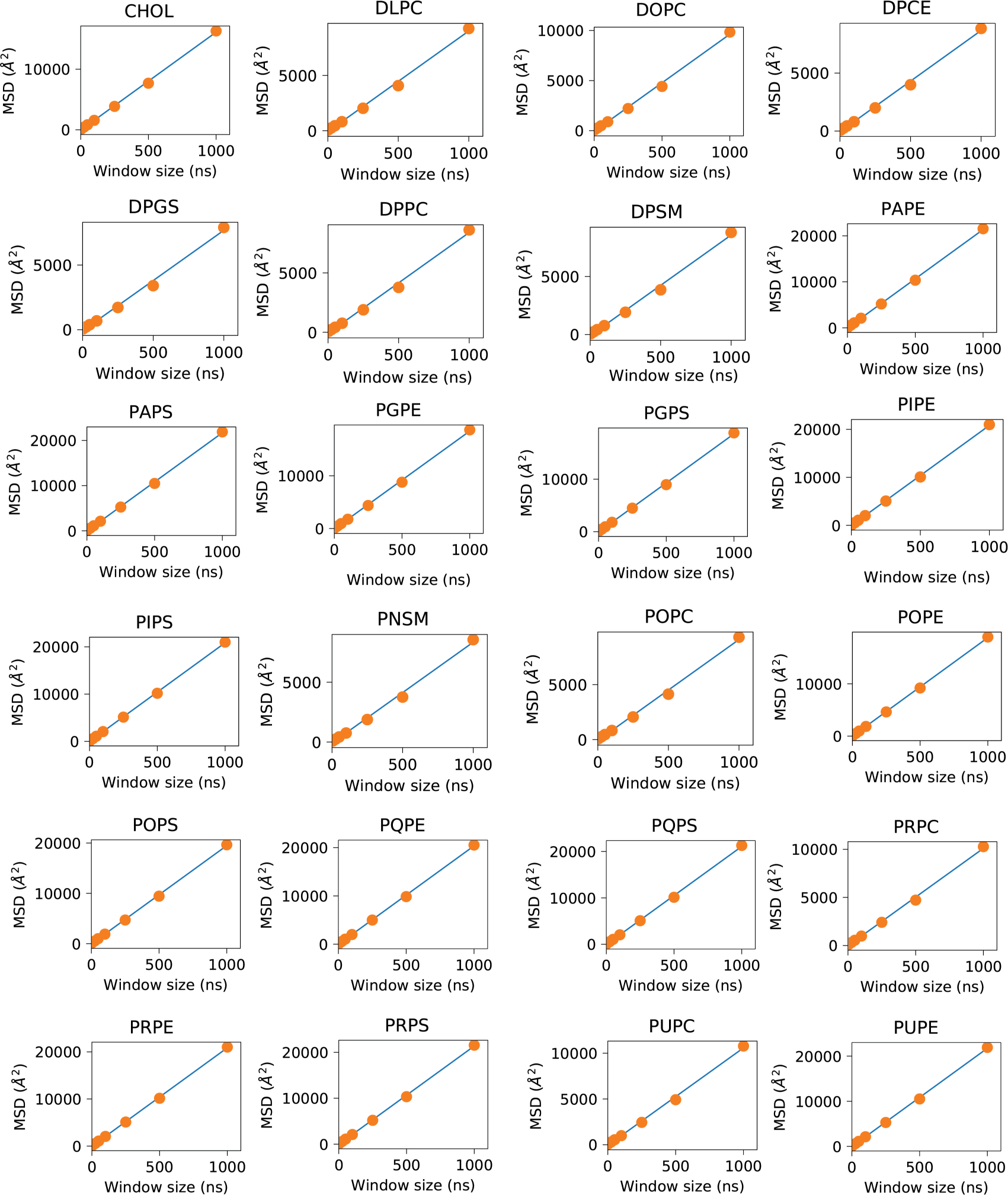

Supplement: S6 Fig — (TIF) [file pcbi.1009781.s006.tif]

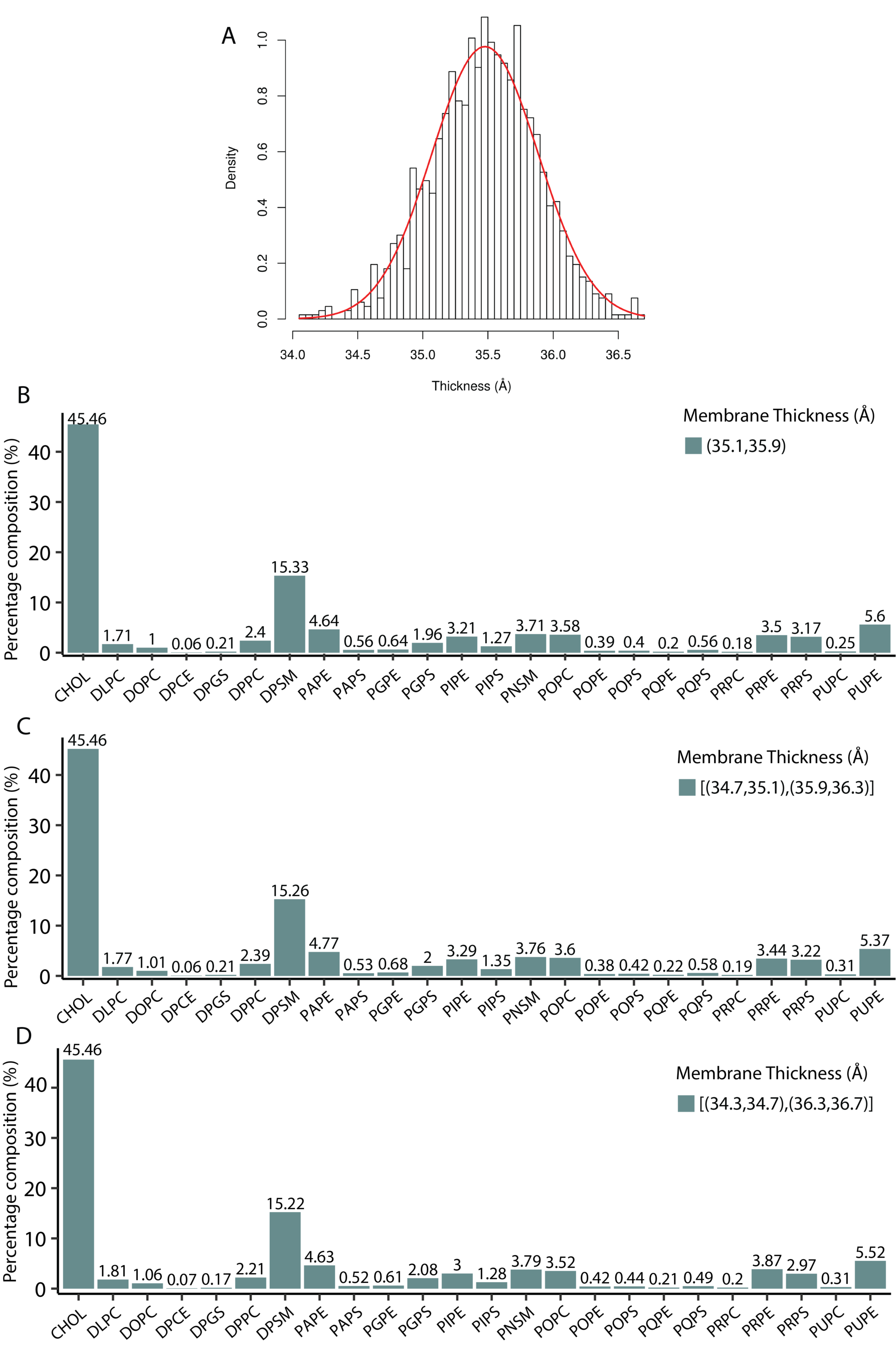

Supplement: S7 Fig — (TIF) [file pcbi.1009781.s007.tif]

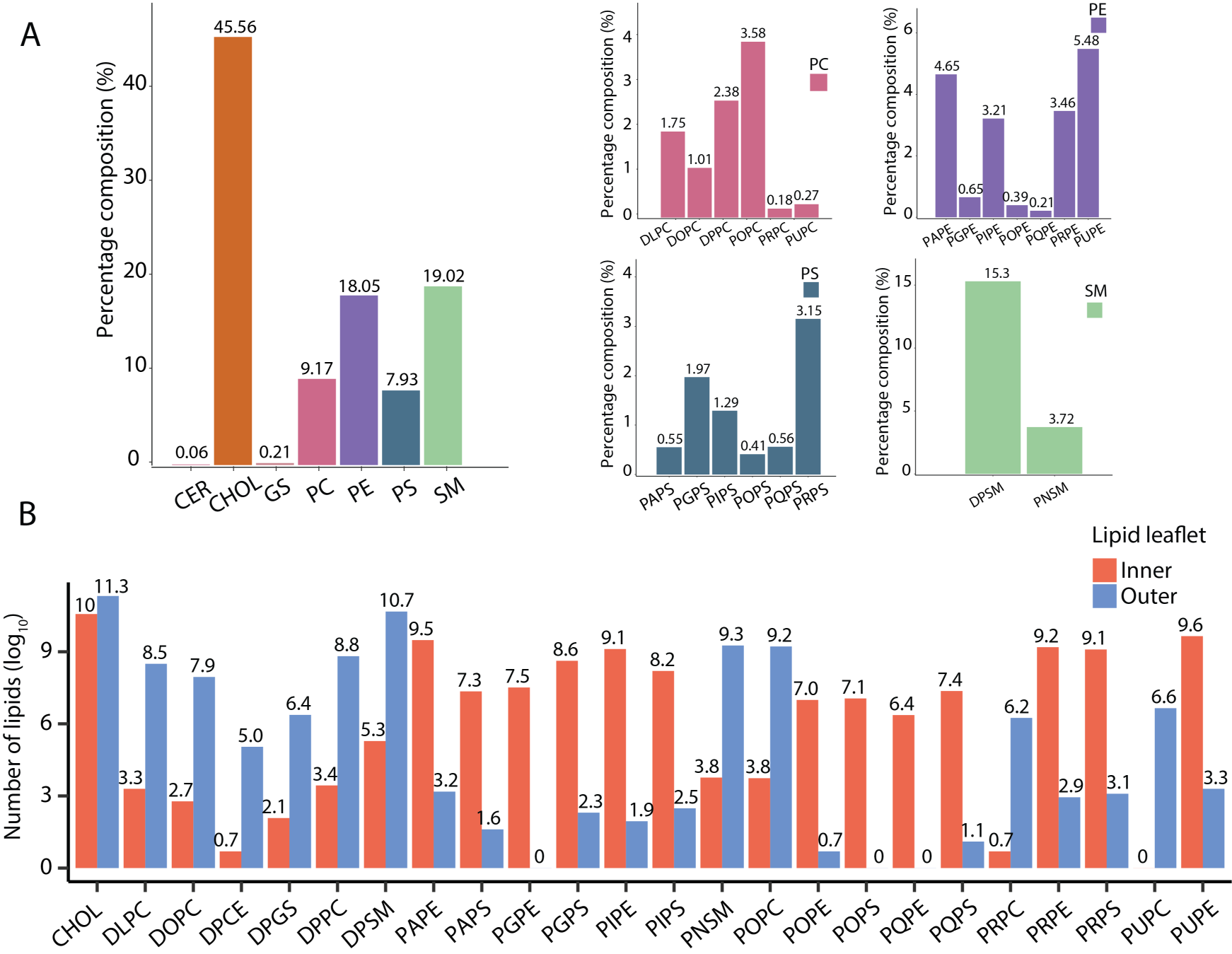

Supplement: S8 Fig — (TIF) [file pcbi.1009781.s008.tif]

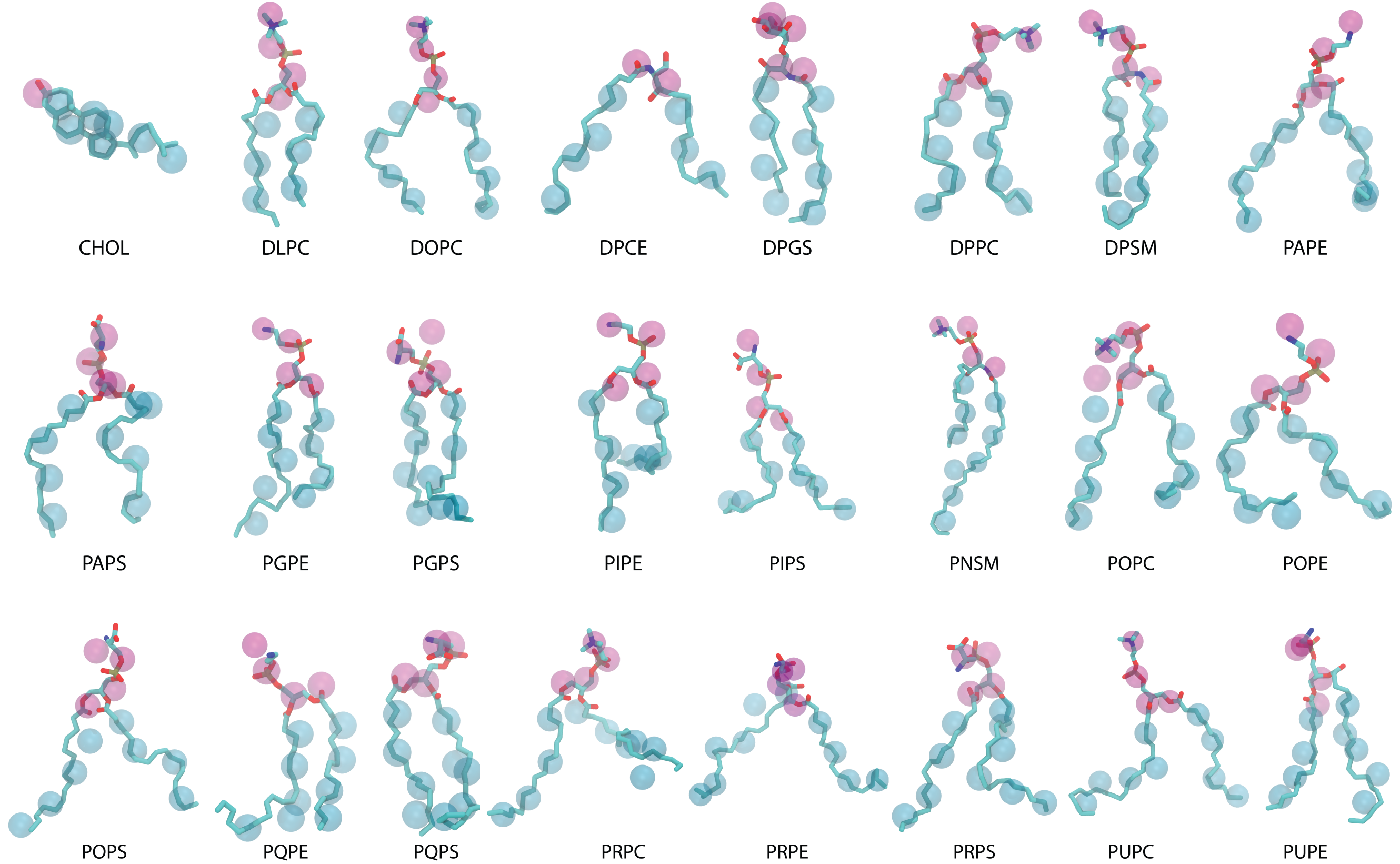

Supplement: S9 Fig — (TIF) [file pcbi.1009781.s009.tif]

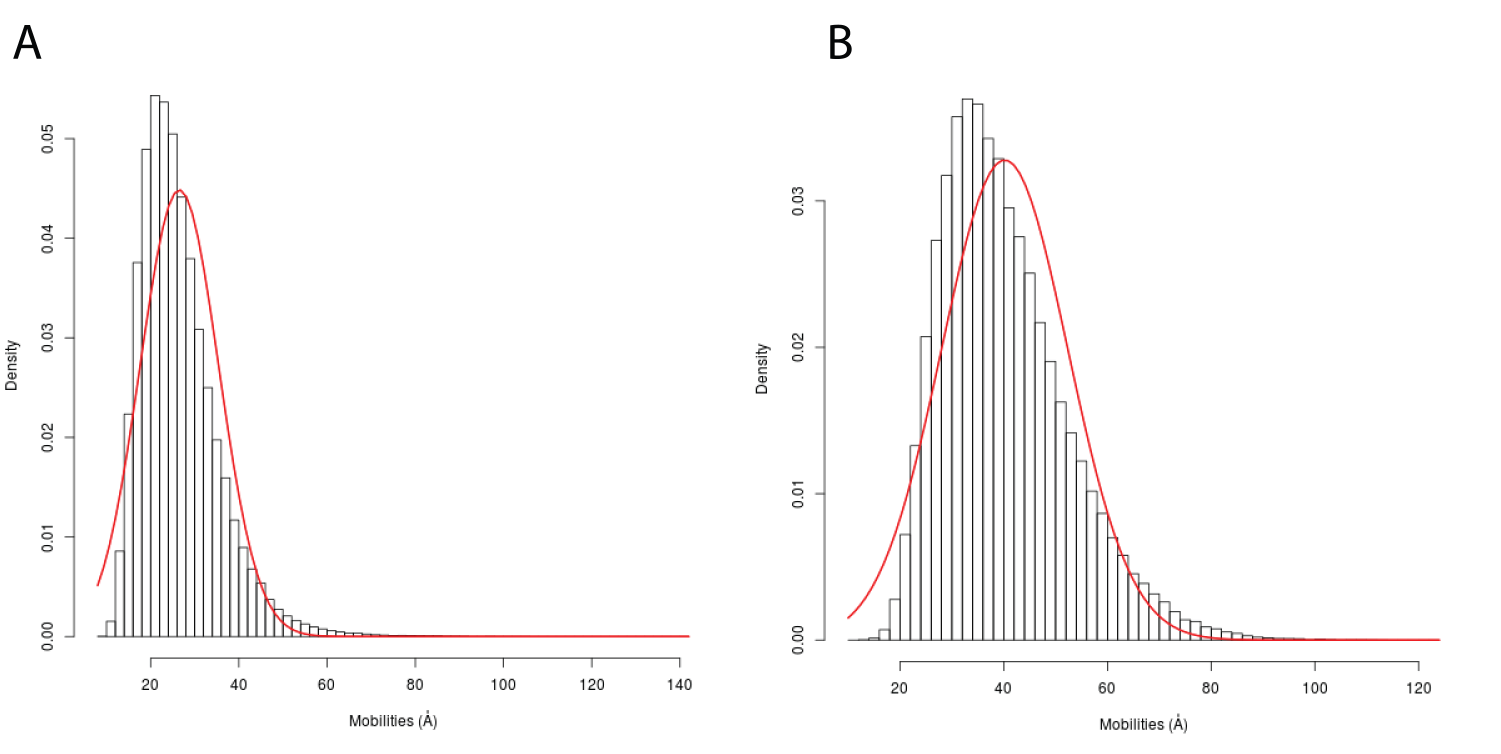

Supplement: S10 Fig — (TIF) [file pcbi.1009781.s010.tif]

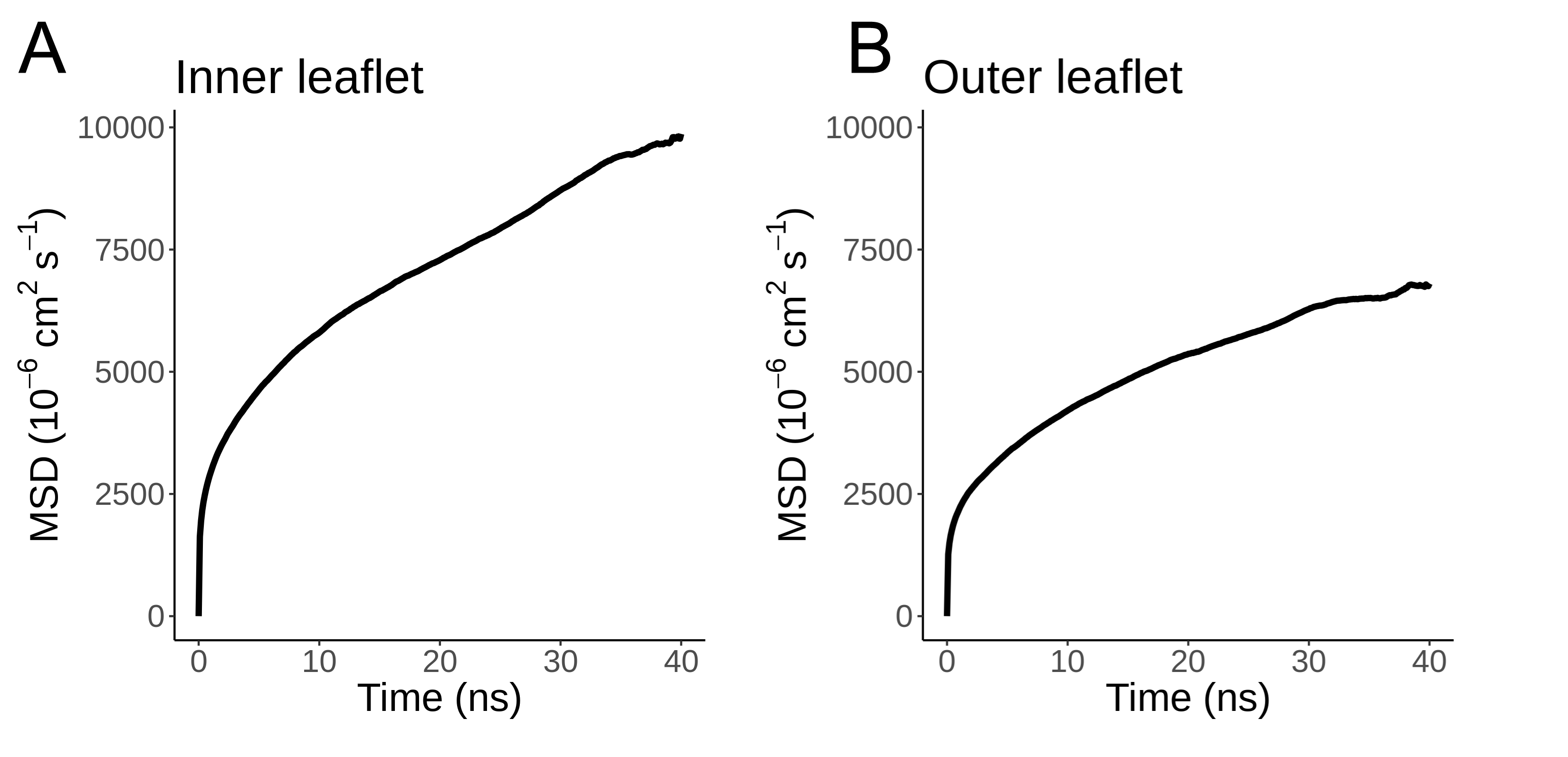

Supplement: S11 Fig — (TIF) [file pcbi.1009781.s011.tif]
