# Supplementary material for: Full scale structural, mechanical and dynamical properties of HIV-1 liposomes
Source: PLoS Comput Biol. 2022 Jan 18;18(1):e1009781. doi: 10.1371/journal.pcbi.1009781 (PMC8797243; doi:10.1371/journal.pcbi.1009781)
Supplement: S1 Table — (PDF) [file pcbi.1009781.s012.pdf]

Table 1: Summary of HIV-1 vesicle inner leaflet and outer leaflet lipidome. Values represent the number of molecules in the inner and outer layer.

	Time: 0 ns		Time: 5200 ns	
**Lipidome**	**Inner leaflet**	**Outer leaflet**	**Inner leaflet**	**Outer leaflet**
**CHOL**	43390	83481	42612	84618
**DPCE**	0	159	2	157
**DPSM**	194	43140	194	43142
**PGPE**	1833	4	1832	6
**PIPS**	3640	23	3640	22
**POPE**	1091	2	1090	6
**PQPS**	1581	9	1581	9
**PRPS**	8912	54	8909	72
**DLPC**	25	4904	25	4904
**DPGS**	7	585	7	585
**PAPE**	13163	59	13159	77
**PGPS**	5572	17	5569	20
**PNSM**	42	10500	42	10500
**POPS**	1160	4	1160	4
**PRPC**	2	516	2	516
**PUPC**	1	775	1	775
**DOPC**	13	2842	13	2842
**DPPC**	29	6725	29	6725
**PAPS**	1557	7	1555	6
**PIPE**	9081	34	9077	36
**POPC**	34	10094	34	10094
**PQPE**	582	4	582	4
**PRPE**	9808	61	9805	59
**PUPE**	15516	64	15507	101
