# Supplementary material for: Full scale structural, mechanical and dynamical properties of HIV-1 liposomes
Source: PLoS Comput Biol. 2022 Jan 18;18(1):e1009781. doi: 10.1371/journal.pcbi.1009781 (PMC8797243; doi:10.1371/journal.pcbi.1009781)
Supplement: S2 Table — (PDF) [file pcbi.1009781.s013.pdf]

Table 1: Chemical names and MARTINI residue names of the lipids used for the vesicle model

**Martini lipid name**	**Chemical name**
**CHOL**	Cholesterol
**DLPC**	1,2-Dilauroyl-sn-glycero-3-phosphocholine
**DOPC**	1,2-Dioleoyl-sn-Glycero-3-Phosphocholine
**DPCE**	N-oleoyl-D-erythro-sphinganine
**DPGS**	N-palmitoyl-D-erythro-glucoceramide
**DPPC**	1,2-dipalmitoyl-sn-glycero-3-phosphocholine
**DPSM**	N-stearoyl-D-erythro-sphingosylphosphorylcholine
**PAPE**	1-palmitoyl-2-arachidonoyl-sn-glycero-3-phosphoethanolamine
**PAPS**	1-palmitoyl-2-arachidonoyl-sn-glycero-3-phospho-L-serine
**PGPE**	1-palmitoyl-2-docosenoyl-sn-glycero-3-phosphoethanolamine
**PGPS**	1-palmitoyl-2-docosenoyl-sn-glycero-3-phospho-L-serine
**PIPE**	1-palmitoyl-2-linoleoyl-sn-glycero-3-phosphoethanolamine
**PIPS**	1-palmitoyl-2-linoleoyl-sn-glycero-3-phospho-L-serine
**PNSM**	N-nervonoyl-D-erythro-sphingosylphosphorylcholine
**POPC**	1-palmitoyl-2-oleoyl-glycero-3-phosphocholine
**POPE**	1-palmitoyl-2-oleoyl-sn-glycero-3-phosphoethanolamine
**POPS**	1-palmitoyl-2-oleoyl-sn-glycero-3-phospho-L-serine
**PQPE**	1-meadoyl-2-palmitoyl-sn-glycero-3-phosphoethanolamine
**PQPS**	1-meadoyl-2-palmitoyl-sn-glycero-3-phospho-L-serine
**PRPC**	1-Palmitoyl-2-lignoceroyl-sn-glycero-3-phosphocholine
**PRPE**	1-Palmitoyl-2-lignoceroyl-sn-glycero-3-phosphoethanolamine
**PRPS**	1-palmitoyl-2-docosahexaenoyl-sn-glycero-3-phospho-L-serine
**PUPC**	1-palmitoyl-2-docosahexaenoyl-sn-glycero-3-phosphocholine
**PUPE**	1-palmitoyl-2-docosahexaenoyl-sn-glycero-3-phosphoethanolamine
