# Supplementary material for: Full scale structural, mechanical and dynamical properties of HIV-1 liposomes
Source: PLoS Comput Biol. 2022 Jan 18;18(1):e1009781. doi: 10.1371/journal.pcbi.1009781 (PMC8797243; doi:10.1371/journal.pcbi.1009781)
Supplement: S3 Table — (PDF) [file pcbi.1009781.s014.pdf]

Composition	Leaflet	
	Intravirion	Exovirion
Asymmetric	103.1±34.6	154.5±33.3
Symmetric, intravirion	189.9±32.9	189.9±32.9
Symmetric, exovirion	205.3±27.8	205.3±27.8

Table 1: Compressibility moduli in units of mN/m from mechanical analysis of flat HIV-1 membrane systems. For the two symmetric systems, which were constructed and equilibrated for 1 μs in order construct the asymmetric flat patch, the presence of 0.5 kJ/mol headgroup restraints leads to higher compression energy.
